# Polarizing the Neuron through Sustained Co-expression of Alternatively Spliced Isoforms

**DOI:** 10.1016/j.celrep.2016.04.012

**Published:** 2016-04-28

**Authors:** Karen Yap, Yixin Xiao, Brad A. Friedman, H. Shawn Je, Eugene V. Makeyev

**Affiliations:** 1 MRC Centre for Developmental Neurobiology, King’s College London, London SE1 1UL, UK; 2 Molecular Neurophysiology Laboratory, Signature Program in Neuroscience and Behavioral Disorders, Duke NUS Graduate Medical School, 8 College Road, 169857 Singapore, Singapore; 3 Department of Physiology, Yong Loo Lin School of Medicine, National University of Singapore, 117597 Singapore, Singapore; 4 Department of Bioinformatics and Computational Biology, Genentech, South San Francisco, CA 94080, USA; 5 School of Biological Sciences, Nanyang Technological University, 637551 Singapore, Singapore

## Abstract

Alternative splicing (AS) is an important source of proteome diversity in eukaryotes. However, how this affects protein repertoires at a single-cell level remains an open question. Here, we show that many 3′-terminal exons are persistently co-expressed with their alternatives in mammalian neurons. In an important example of this scenario, cell polarity gene *Cdc42*, a combination of polypyrimidine tract-binding, protein-dependent, and constitutive splicing mechanisms ensures a halfway switch from the general (E7) to the neuron-specific (E6) alternative 3′-terminal exon during neuronal differentiation. Perturbing the nearly equimolar E6/E7 ratio in neurons results in defects in both axonal and dendritic compartments and suggests that Cdc42E7 is involved in axonogenesis, whereas Cdc42E6 is required for normal development of dendritic spines. Thus, co-expression of a precise blend of functionally distinct splice isoforms rather than a complete switch from one isoform to another underlies proper structural and functional polarization of neurons.

## Highlights

• Alternative 3′-terminal isoforms often become co-expressed in developing neurons
• Ptbp1/2-dependent and constitutive mechanisms ensure co-expression of Cdc42 isoforms
• Controlled utilization of Cdc42 exon 7 in neurons is required for proper axonogenesis
• Exon-6-containing isoform of Cdc42 promotes formation of dendritic spines in vivo

## Graphical Abstract

**Figure:**
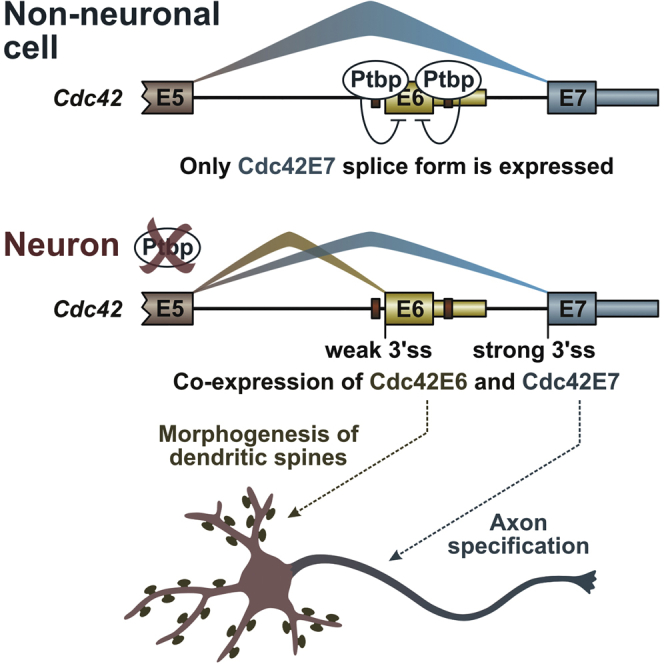

## Teaser

Alternative splicing diversifies eukaryotic proteomes, but how this affects protein functions in individual cells remains poorly understood. Yap et al. show that polypyrimidine tract-binding, protein-dependent, and constitutive mechanisms ensure co-expression of two alternative isoforms of Cdc42 in neurons. Importantly, this is required for proper development of axons and dendritic spines.

## Introduction

Alternative pre-mRNA splicing (AS) provides an efficient means for maximizing the protein-coding capacity of eukaryotic genomes and a likely source of progressive evolutionary elaboration in the metazoan clade (Maniatis and Tasic, 2002, Nilsen and Graveley, 2010). Many AS events give rise to tissue- or developmental-stage-specific protein isoforms, which may facilitate morphological and functional differentiation of corresponding cell types (Kalsotra and Cooper, 2011, Pan et al., 2008, Wang et al., 2008).

A few cases have been reported where AS could result in sustained co-expression of functionally distinct isoforms in individual cells. Perhaps the most striking example is the *Drosophila* Dscam1 surface protein promoting homophilic repulsion of neurites originating from the same neuron (Hattori et al., 2008). The *Dscam1* gene contains extensive arrays of mutually exclusive cassette exons that can be spliced in a combinatorial manner to generate up to 38,016 distinct protein variants (Park and Graveley, 2007). The exons are selected in an apparently random manner, and each individual neuron is thought to co-express a unique blend of 10–30 Dscam1 isoforms distinguishing it from its neighbors (Miura et al., 2013, Zhan et al., 2004).

In the mammalian brain, AS of neurexin transcripts gives rise to hundreds of mRNA products, with individual neurons potentially co-expressing several distinct isoforms (Fuccillo et al., 2015, Schreiner et al., 2014). Neurexins play a critical role in synapse assembly and functional differentiation by interacting with their post-synaptic partners (Craig and Kang, 2007, Williams et al., 2010). This suggests that the AS-generated molecular diversity might provide a surface code for integration of individual neurons into larger circuits (Fuccillo et al., 2015, Schreiner et al., 2014, Williams et al., 2010).

Could co-expression of functionally distinct isoforms in the same cell represent a more general function of AS? Published transcriptome-wide analyses suggest that many genes might generate a mixture of isoforms in specific mammalian tissues (Pan et al., 2008, Wang et al., 2008). However, tissues are composed of different types of mature cells, their progenitors, and differentiation intermediates, which makes it generally unclear whether isoforms present in the same sample co-occur at the cellular level. Rapidly developing single-cell RNA sequencing (RNA-seq) techniques (Wang and Navin, 2015) should tackle this problem but, as such, are not expected to provide insights into functional importance of co-expressed transcripts.

A common type of AS involves a choice between two or more alternative 3′-terminal exons (A3Es) that often modulate domain composition and C-terminal structure of protein products (Kelemen et al., 2013, Nilsen and Graveley, 2010, Zheng and Black, 2013). One example of this regulation is the *Cdc42* gene that encodes a Rho family GTPase essential for normal actin cytoskeleton dynamics, cell projection outgrowth, and cell polarity (Govek et al., 2005, Melendez et al., 2011, Tahirovic and Bradke, 2009). Alternative utilization of two A3Es, exons 6 (E6) and 7 (E7), gives rise to corresponding Cdc42 variants with distinct C termini (Chen et al., 2012). Of these, E7 is utilized ubiquitously, while E6 is upregulated in the nervous system by poorly understood mechanisms. It is currently unclear whether neurons express only E6-terminated or a mixture of the E6- and E7-terminated isoforms.

Both E6 and E7 encode CAAX-box motifs that are post-translationally prenylated, whereas the E6-specific amino acid sequence can be additionally palmitoylated (Kang et al., 2008, Nishimura and Linder, 2013, Wirth et al., 2013). The latter modification accounts for preferential localization of the E6-terminated protein isoform to dendritic spines and its role in morphogenesis of these post-synaptic structures (Kang et al., 2008). Interestingly, conditional inactivation of the *Cdc42* gene in cortical neurons reduces the efficiency of axon formation (Garvalov et al., 2007). However, which of the two isoforms is responsible for this activity and how the balance between axonal and dendritic activities of Cdc42 is maintained remain poorly understood.

Here, we took a bioinformatics approach to identify genes potentially co-expressing functionally important A3E assortments in mammalian neurons. We reasoned that this category might be characterized by a monotonic developmental transition from a single A3E isoform in progenitor cells to a mixture of isoforms stably expressed in terminally differentiated neurons. Using a quantitative metric of isoform co-expression, we show that a substantial fraction of genes, indeed, follows this trend, and their subset regulated by polypyrimidine tract-binding proteins (Ptbps) Ptbp1 and Ptbp2 (Keppetipola et al., 2012) is enriched for GTP binding and membrane-associated regulators of cellular projections. In an important example of this regulation, a switch from the exclusive production of the general isoform Cdc42E7 in neuronal precursors and non-neuronal cells to stable co-expression of the Cdc42E6 and Cdc42E7 isoforms at a single-neuron level is orchestrated through developmental changes in Ptbp1 and Ptbp2 abundance and a constitutive difference in the relative strengths of the E6 and E7 splicing acceptor sequences. We further show that the two co-expressed isoforms are functionally specialized in neurons and uncover an unexpected function of the Cdc42E7 protein in axonogenesis. Taken together, these results advance our understanding of mechanisms underlying axo-dendritic polarity in developing neurons and argue that co-expression of functionally distinct AS isoforms in the same cell is a more prevalent and biologically important scenario than previously thought.

## Results

### Alternative 3′-Terminal Exons Are Extensively Regulated in Developing Neurons

**Figure 1:**
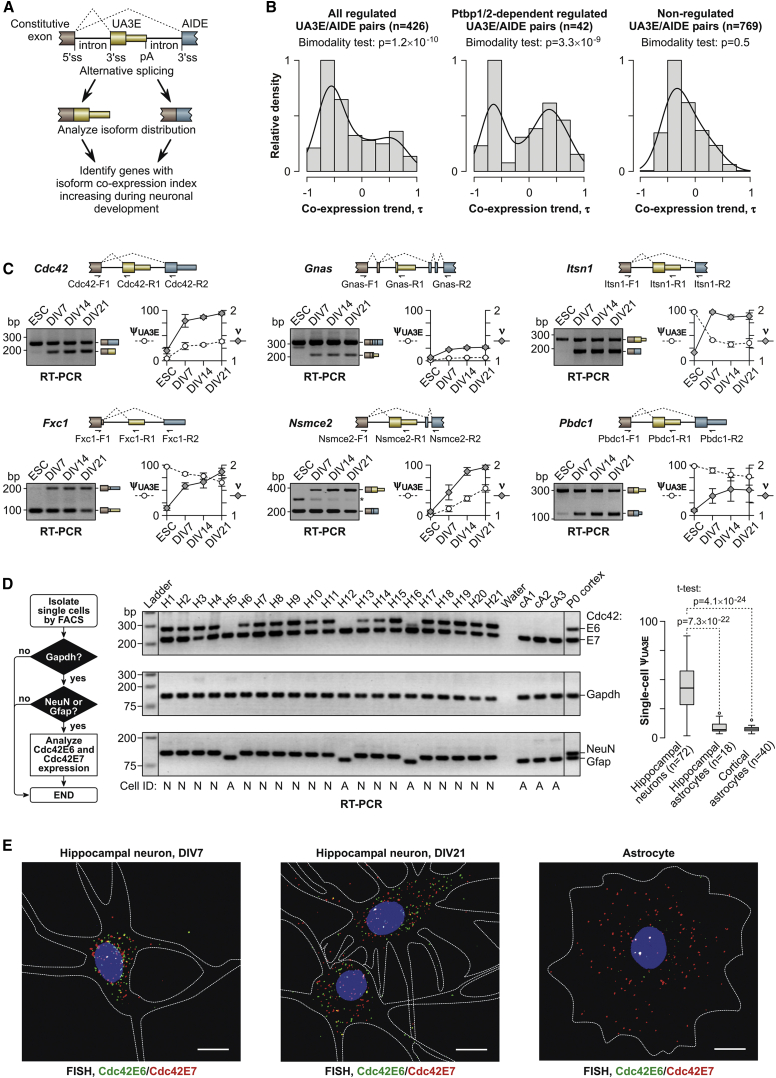
Co-expression of Alternative 3′-Terminal Exons in Developing Neurons (A) Bioinformatics approach used to identify pairs of UA3Es and corresponding AIDEs that are increasingly co-expressed during neuronal development. (B) Distributions of Kendall rank correlation coefficients (τ) of time-resolved ν trajectories for all (n = 426) and Ptbp1/2-dependent (n = 42) regulated UA3E/AIDE pairs, as well as for non-regulated events (n = 769). Shown are histograms normalized to the height of the most populated bin and the corresponding distribution density estimates. Note that the distributions are bimodal for both regulated subsets, with the two peaks corresponding to UA3E/AIDE pairs with increasing and decreasing co-expression trends, respectively. (C) Ptbp1/2-dependent (Cdc42, Gnas, and Itsn 1) and -independent (Fxc1, Nsmce2, and Pbdc1) UA3E/AIDE pairs with increasing co-expression trends were analyzed by multiplex RT-PCR in mouse ESCs and primary hippocampal neurons at different stages of maturation (DIV7–DIV21). Relevant gene fragments and PCR primers used for the analysis are depicted on the upper panel, whereas corresponding gel images and Δψ_UA3E_ and ν quantitations are shown at the lower panel. Data are averaged from three experiments using independent cell cultures ± SE. (D) Single-cell analysis of Cdc42 isoform co-expression. As summarized in the flowchart on the left, cells were acutely isolated from newborn mouse hippocampi by FACS and assayed by RT-PCR for housekeeping (Gapdh), neuronal (NeuN), and astroglial (Gfap) markers prior to estimating proportional abundance of the Cdc42E6 and Cdc42E7 isoforms. The three RT-PCR panels in the middle show representative analyses of 21 hippocampal cells (H1–H21) identified as neurons (NeuN^+^/Gfap^−^; N) or astrocytes (NeuN^−^/Gfap^+^; A). Note that all hippocampal neurons express comparable amounts of Cdc42E6 and Cdc42E7, whereas hippocampal astrocytes express almost exclusively Cdc42E7. A similar preference for Cdc42E7 was detected in astrocytes enriched from newborn mouse cortex (cells cA1–cA3). As a control, we additionally analyzed total RNA extracted from an entire mouse cortex (lane “P0 cortex”). A boxplot quantitation of the Cdc42 isoform expression in the entire single-cell RT-PCR dataset collected for 72 hippocampal neurons, 18 hippocampal astrocytes, and 40 cortical astrocytes is shown on the right. Samples were compared by two-tailed t test, assuming unequal variance. (E) Individual mRNA molecule-resolution RNA FISH analyses showing that hippocampal neurons persistently co-express comparable amounts of the two Cdc42 isoforms from DIV7 through DIV21, whereas astrocytes express almost exclusively Cdc42E7. Scale bars, 10 μm. White dashed lines show cell contours. See also Figures S1 and S2.

We cataloged “upstream A3Es” (UA3Es) mapping within an intron of an alternative isoform containing downstream exon(s) (AIDE) (Figure 1A) and examined the utilization of this category in mouse embryonic stem cells (ESCs) differentiating into highly homogeneous populations of glutamatergic neurons (GNs; GN1 through GN5, in order of maturity) through neural stem cell (NSC) and radial glia cell (RGC) intermediates (Hubbard et al., 2013) (Figures S1A and S1B). Of the 1,481 annotated UA3Es, 1,195 were detectable by RNA-seq, at least at one differentiation stage, and 426 underwent significant splicing changes (see Experimental Procedures for more detail). Interestingly, analysis of the 250-nt sequence window centered on the U3AE 3′ splice sites (3′ss; regions R4 and R5 in Figure S1C) revealed a significant over-representation of several pyrimidine-rich RNA-binding protein (RBP) motifs in the 426 regulated UA3Es compared to the 769 non-regulated ones (Table S1; Supplemental Experimental Procedures).

Ptbp1 and its neuronal paralog Ptbp2 are global regulators of the nervous system-specific AS program (Keppetipola et al., 2012). Ptbp1 is downregulated on the onset of neurogenesis by brain-enriched microRNA miR-124 (Makeyev et al., 2007), whereas Ptbp2 expression transiently peaks in young neurons and subsequently declines in mature ones (Zheng et al., 2012). Since these trends were obvious in developing GNs (Figure S1B) and Ptb protein-specific motifs were enriched in the 3′ss-adjacent sequences of the regulated UA3Es (Figures S1D and S1E; Table S1), we wondered whether Ptbp1 and Ptbp2 could contribute to the UA3E regulation.

To this end, we turned to CAD neuroblastoma cells known to express Ptbp1 at readily detectable levels and upregulate Ptbp2 upon Ptbp1 knockdown (Makeyev et al., 2007, Yap et al., 2012). RNA-seq analysis of CAD cells treated with small interfering RNAs (siRNAs) against Ptbp1 or both Ptbp1 and Ptbp2 showed that, of the 1,195 UA3Es expressed in the GN differentiation model, 65 were consistently regulated by Ptbp1 and Ptbp2 (Supplemental Experimental Procedures). Notably, 42 Ptbp1/2-dependent UA3Es were also present within the 426 developmentally regulated exons, a significant enrichment according to Fisher’s exact test (p = 4.8 × 10^−3^). Moreover, sequences immediately preceding and following the 3′ss in this 42-UA3E cohort were enriched in Ptb protein consensus motifs considerably stronger than in the entire set of 426 regulated exons (Figures S1E and S1F).

We concluded that multiple UA3E/AIDE pairs change their splicing patterns during neuronal differentiation, and a substantial fraction of these events might be regulated in a Ptbp1/2-dependent manner.

### Many Genes Tend to Increase Isoform Co-expression in Developing Neurons

To facilitate further analyses, we introduced the isoform co-expression index (ν) based on a statistic describing the effective diversity of species in a sample (Hill, 1973)

ν=exp(H).

Here, *H* is Shannon’s entropy calculated for a given isoform mixture, with proportional abundance of each isoform characterized by its “percent-spliced-in” value (ψ; Wang et al., 2008). For two AS possibilities, UA3E and AIDE, ν assumes its maximal value of 2 if ψ_UA3E_ = ψ_AIDE_ = 50%, and both mRNA species are expected to give rise to substantial amounts of protein products. Conversely, ν approaches its minimum, 1, when one isoform is markedly less abundant than the other (ψ_UA3E_ < < ψ_AIDE_ or ψ_UA3E_ ≫ ψ_AIDE_) and, thus, unlikely to have a biologically relevant translational output (Supplemental Experimental Procedures). Thus, ν may provide a useful proxy of co-expression of both isoforms at functionally relevant levels.

To identify possible situations where the two isoforms were increasingly co-expressed during neuronal development, we calculated Kendall rank correlation coefficients (τ; Hipel and McLeod, 1994) between ν and developmental time for each UA3E/AIDE pair. Interestingly, τ was distributed bimodally for all 426 regulated UA3E/AIDEs and the Ptbp1/2-dependent 42-exon subset with the two peaks containing positive and negative values (Figure 1B; bimodality test p values = 1.2 × 10^−10^ and 3.3 × 10^−9^, respectively) (Holzmann and Vollmer, 2008). On the other hand, τ was unimodal for the 769 non-regulated UA3E/AIDEs (Figure 1B; bimodality test p value = 0.5). A closer inspection of the positive τ peaks showed that the isoform co-expression index for 81 of the total 426 regulated UA3E/AIDEs and ten of the 42 Ptbp1/2-dependent events significantly increased as a function of development (τ > 0.4; Benjamini-Hochberg (BH)-adjusted Kendall test p value < 0.005; Table S2).

These data suggested that isoform co-expression frequently undergoes directional changes during neuronal differentiation, and a distinct subset of regulated AS events might favor co-expression of UA3E and AIDE in neurons.

### Developmental Increase in Isoform Co-expression Is Also Apparent in Primary Cells

To test whether genes with developmentally increasing UA3E/AIDE co-expression trends behaved similarly in primary cells, we analyzed their splicing patterns in mouse ESCs, cortical NSCs (cNSCs), and cortical neurons at different stages of maturation (days in vitro [DIV] 0 to DIV31) using multiplex RT-PCR (Figure S2A). All Ptbp1/2-dependent and -independent genes assayed in this manner showed ψ_UA3E_ and ν trajectories similar to those predicted for in-vitro-derived GNs. Moreover, ν tended to plateau at relatively high values at later stages of neuronal maturation (Figure S2A). Similar trends were also apparent in primary hippocampal neurons (Figure 1C). Importantly, cNSC and primary neuronal cultures used in these analyses were highly homogeneous (Figure S2B), thus confirming that the UA3E- and the AIDE-terminated splice forms were, indeed, co-expressed in the corresponding cell types.

This argued that many UA3E/AIDE pairs become co-expressed in neurons, potentially giving rise to biologically relevant amounts of corresponding protein isoforms.

### Functional Enrichment of Ptbp1/2-Dependent Genes Co-expressing UA3E and AIDE in Neurons

Gene ontology (GO) analysis of Ptbp1/2-dependent UA3E/AIDE pairs with increasing overexpression trends uncovered their significant enrichment for cell projection, plasma membrane association, and GTP-binding/hydrolysis terms (Benjamini-adjusted p < 0.05) (Table S3). On the other hand, no significant functional enrichment was detected for the rest of the Ptbp1/2-regulated events (Benjamini-adjusted p ≥ 0.36). All genes shortlisted by the GO analysis (*Cdc42*, *Itsn1*, *Gnas*, *Dnm2*, *Ncam1*, and *Sept11*) had documented neuronal functions (Bromberg et al., 2008, Ditlevsen et al., 2008, González-Jamett et al., 2014, Govek et al., 2005, Li et al., 2009, Melendez et al., 2011, Pechstein et al., 2010, Tahirovic and Bradke, 2009) and were predicted to produce A3E isoforms with markedly different C-terminal amino acid sequences. Ptbp1/2 dependence of the choice between UA3E and AIDE in five of these genes was confirmed by multiplex RT-PCR analyses of CAD cells treated with control, Ptbp1-specific, or both Ptbp1- and Ptbp2-specific siRNAs (siControl, siPtbp1, or siPtbp1/2, respectively; Figures S2C–S2E).

This indicated that downregulation of Ptbp1 and, subsequently, Ptbp2 during neuronal development might diversify the isoform repertoires of functionally important genes.

### Hippocampal Neurons, but not Astrocytes, Co-express Exon-6- and Exon-7-Terminated Isoforms of Cdc42

To address the biological significance of UA3E/AIDE co-expression, we focused on the *Cdc42* gene representing all enriched GO categories (Table S3). First, we wanted to confirm that Cdc42 isoforms terminated with either neuron-enriched E6 (the UA3E) or ubiquitous E7 (the AIDE) were, indeed, co-expressed in the same neuron. To this end, individual cells from newborn mouse hippocampi were analyzed by RT-PCR using cell-type-specific and Cdc42-specific primer mixtures (Figure 1D). Neurons identified by the presence of neuronal mRNA NeuN (also known as Rbfox3) and the absence of astroglial mRNA Gfap (NeuN^+^/Gfap^−^ cells) constituted 58.54% of the total population, whereas astrocytes (NeuN^−^/Gfap^+^ cells) made up 14.63%. The remaining 26.83% of cells expressing the “housekeeping” gene *Gapdh* but showing no conclusive expression of either NeuN or Gfap likely belonged to other lineages and were not analyzed further. Notably, all NeuN^+^/Gfap^−^ neurons expressed readily detectable amounts of both Cdc42E6 and Cdc42E7 with the ψ_UA3E_ median value of 42.8% (Figure 1D). On the other hand, NeuN^−^/Gfap^+^ astrocytes expressed almost exclusively Cdc42E7 (ψ_UA3E_ median, 4.5%; Figure 1D). Similar preference for Cdc42E7 was detected in NeuN^−^/Gfap^+^ astrocytes enriched from newborn mouse cortex (ψ_UA3E_ median, 4.9%; Figure 1D).

We additionally investigated isoform co-expression using dual-color RNA fluorescence in situ hybridization (FISH) using corresponding probe sets (Figure 1E). This experiment confirmed that neurons at different stages of maturation (DIV7 and DIV21) co-express Cdc42E6 and Cdc42E7 at comparable levels, whereas astrocytes express almost exclusively Cdc42E7 (Figure 1E).

Thus, neurons, but not astrocytes, persistently co-express Cdc42E6 and Cdc42E7 at the individual cell level.

### Ptbp1 and Ptbp2 Repress Cdc42 E6 by Interacting with Corresponding *cis*-Elements

**Figure 2:**
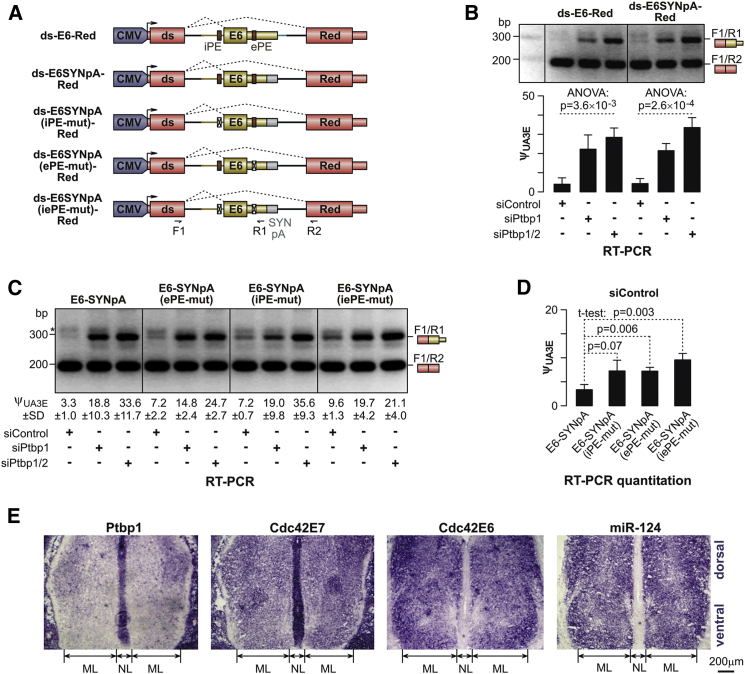
E6 of Cdc42 Pre-mRNA Is an Example of UA3E Regulated by Ptbps (A) dsRed-based minigenes containing Cdc42 E6 with its natural or synthetic polyadenylation context and WT or mutated clusters of pyrimidine-rich elements (iPE and ePE). Arrows indicate primers used for multiplex RT-PCR analyses. (B) CAD cells pretreated for 48 hr with siControl, siPtbp1, or siPtbp1/2 were transfected for another 24 hr with either ds-E6-Red or ds-E6SYNpA-Red, and minigene-specific splicing patterns were analyzed by multiplex RT-PCR with F1/R1/R2 primers. Note that knockdown of Ptbp1 alone, and especially in combination with Ptbp2, stimulates utilization of Cdc42 E6 for both minigenes in a manner virtually indistinguishable from that of endogenous Cdc42 transcripts (Figure S2E). Upper: agarose gel analyses of the RT-PCR products. Lower: E6-specific percent-spliced-in values (ψ_UA3E_). (C) Multiplex RT-PCR analysis of the effect of iPE and/or ePE mutations introduced in (A) on AS of minigene transcripts. (D) Quantitative comparison of ψ_UA3E_ values between WT and mutant versions of E6, showing that both PE mutations stimulate E6 inclusion in siControl samples. (E) Alkaline phosphatase in situ hybridization analyses of embryonic-day (E)13.5 developing mouse neural tube sectioned at the hindbrain level and stained with digoxigenin-labeled RNA probes against Ptbp1 or either of the two Cdc42 isoforms, Cdc42E6 or Cdc42E7. Also shown is staining for miR-124 with a complementary digoxigenin-labeled locked nucleic acid (LNA) probe (Makeyev et al., 2007). Note that Ptbp1 is expressed at a high level in mesenchymal cells surrounding the neural tube and the two closely opposed neuroepithelial layer (NL) sheets lining the fourth ventricle and containing NSCs. As expected (Makeyev et al., 2007), miR-124 downregulates Ptbp1 in the mantle layer (ML) containing developing neurons. Note that Cdc42E7 is expressed in the Ptbp1-positive regions at a relatively high level and in the Ptbp1-depleted ML at a reduced but detectable level, whereas Cdc42E6 expression is restricted to the ML. Data in (B) and (D) are averaged from three independent experiments ± SD and compared using two-tailed t test or one-way ANOVA. See also Figures S2 and S3.

We then wondered what molecular mechanisms could ensure exclusive utilization of E7 in non-neuronal cells and upregulation of E6 in neurons. Sequences adjacent to the junction between intron 5 and E6 contained 18 Ptbp1/2-specific consensus hexamers (Figure S3A), indicating that Ptbp1 and Ptbp2 could regulate E6 by directly interacting with these motifs. To test this hypothesis, E6 and adjacent genomic sequences were inserted into a constitutively spliced intron of a *dsRed* gene (ds-E6-Red; Figure 2A) and expressed in CAD cells pretreated with siControl, siPtbp1, or siPtbp1/2 (Figure 2B). In the siControl samples, E6 was skipped, and the only product detectable by RT-PCR was correctly spliced dsRed mRNA (Figure 2B). However, knockdown of Ptbp1, either alone or together with Ptbp2, stimulated E6 utilization, giving rise to a mixture of dsRed and ds-E6 mRNA species (Figure 2B).

Sequence containing a 3′-terminal part of intron 5 and a 5′-terminal part of E6 and comprising all 18 Ptbp1/2 hexamers was sufficient for this regulation, since ds-E6SYNpA-Red transcripts with a synthetic cleavage/polyadenylation sequence replacing the natural E6 3′ end responded to siPtbp1 and siPtbp1/2 similarly to ds-E6WTpA-Red (Figures 2A and 2B). Importantly, the ds- E7′-Red minigene containing a version of Cdc42 E7 generated an invariant mixture of dsRed and ds- E7′ splicing products in all siRNA-treated samples (Figure S3B).

Interestingly, five intronic and four exonic Ptbp1- and Ptbp2-specific motifs formed relatively compact clusters (iPE and ePE, respectively) (Figure 2A; Figure S3A). Since both iPE and ePE were conserved across mammals (Figure S3A) and interacted with Ptbp1 protein in our in vitro binding assays (Figures S3C–S3H), we tested whether they participated in AS regulation. Mutating these sequence clusters individually (ds-E6SYNpA(iPE-mut)-Red and ds-E6SYNpA(ePE-mut)-Red) promoted E6 inclusion in siControl-treated cells ∼2.2-fold, whereas mutating them simultaneously (ds-E6SYNpA(iePE-mut)-Red) led to ∼2.9-fold stimulation effect (p = 0.003; Figures 2C and 2D).

These results suggested that binding of Ptbp1 and, possibly, Ptbp2 to their cognate *cis*-elements within or near E6 inhibits this exon and limits Cdc42 AS choice in non-neuronal cells to E7. Strongly supporting this model, our in situ hybridization data showed that patterns of Cdc42E6 upregulation and Cdc42E7 downregulation in the developing neural tube matched Ptbp1 dynamics in response to miR-124 (Figure 2E).

### Stronger Splice Acceptor of E7 Ensures Co-expression of Cdc42E6 and Cdc42E7 Isoforms in Neurons

We then wondered why the switch from E7 to E6 in response to reduced Ptbp1/2 activity was only partial in both neurons and siRNA-treated CAD cells. This was especially surprising, since proximal exons are expected to have a substantial advantage over distal ones during both co-transcriptional and post-transcriptional phases of pre-mRNA splicing (Kornblihtt et al., 2013, Reed and Maniatis, 1986). We hypothesized that this might be due to a difference in the strength of constitutive splicing signals between E6 and E7. To test this prediction, we generated the ds-E6-E7 expression construct containing an entire *Cdc42* 3′-terminal fragment downstream of the first exon and the splicing donor sequence of the *dsRed* gene (Figure S3I). This construct recapitulated the incomplete nature of the Cdc42 AS switch in siRNA-treated CAD cells (Figure S3J).

Then, we substituted the splicing acceptor sequence of E6 with its E7 counterpart so that both the iPE and ePE remained intact (Figure S3J). The resultant ds-E7′/E6-E7 transcripts retained their Ptbp1/2 AS dependence, but the choice between the two A3Es was strongly biased toward the upstream E7′/E6 hybrid, with virtually no ds-E7 products detectable in siPtbp1/Ptbp2-treated cells (Figure S3J). Importantly, the reciprocal swap construct, where the splicing acceptor sequence of E7 was replaced with the corresponding E6 fragment comprising both iPE and ePE, converted the upstream wild-type (WT) E6 into a predominant AS choice that did not depend on Ptbp1 and Ptbp2 levels (Figures S3I and S3J).

Thus, the difference in the relative strengths of E6 and E7 splicing acceptor sequences likely ensures co-expression of comparable amounts of the two isoforms in neurons.

### Cdc42E6 and Cdc42E7 Have Distinct Functions in Primary Neurons

**Figure 3:**
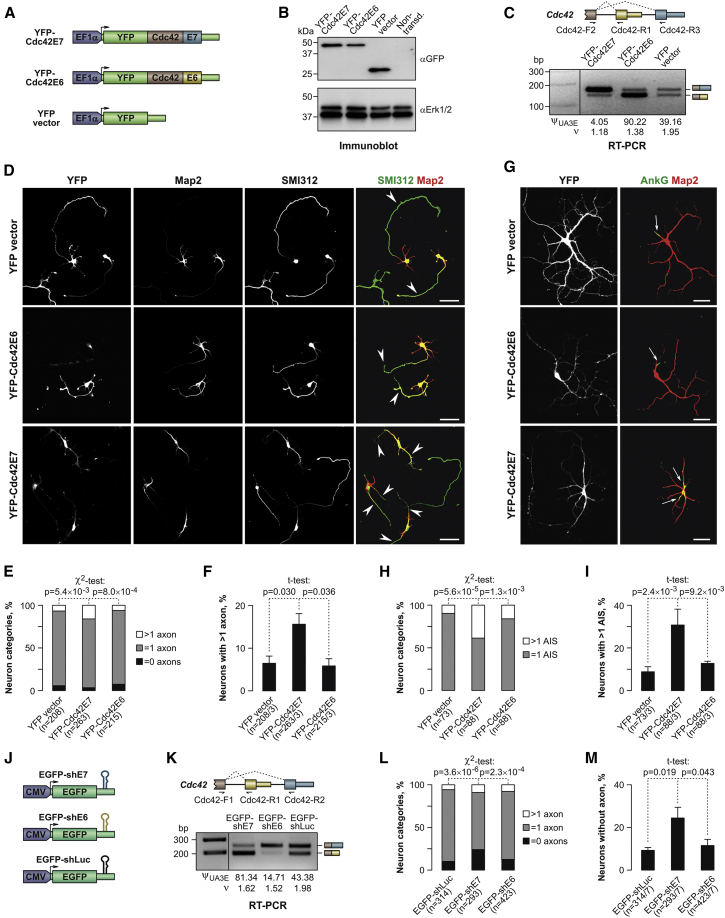
Cdc42E7 Promotes Axonogenesis (A) Lentiviral expression constructs encoding YFP-tagged Cdc42E6 and Cdc42E7 protein isoforms and the corresponding YFP vector control. (B) Neuronal cultures were transduced with the lentiviral constructs shown in (A) at DIV0 and analyzed at DIV3 by immunoblotting with an anti-GFP antibody. An anti-Erk1/2 antibody was used as a lane loading control. Note that the two YFP-tagged Cdc42 isoforms are expressed at comparable levels. (C) RT-PCR analysis of the aforementioned samples with primers recognizing both endogenous and lentivirus-encoded Cdc42 mRNAs confirms that the recombinant Cdc42 isoforms alter the natural E6/E7 balance. Upper: RT-PCR primer annealing sites shown for endogenous *Cdc42*. Lower: agarose gel analysis of the RT-PCR products. (D–I) Immunostaining of hippocampal neurons transduced with the constructs in (A) reveals a higher incidence of cells with more than one axon in Cdc42E7-expressing samples as compared to Cdc42E6 and YFP. (D) Representative DIV3 neurons stained for the axonal marker SMI312 and dendritic marker Map2. (E) χ^2^ test analysis of neuronal categories with zero, one, and more than one axon in (D). (F) t test comparisons of neuronal fractions containing more than one SMI312-positive axon in (D). (G) Representative DIV14 neurons stained for the AIS marker AnkG. (H) χ^2^ test comparison of neurons with one and more than one AIS in (G). (I) Percentage of neurons in (G) with more than one AIS compared by t test. Scale bars, 50 μm in (D) and (G). (J) Lentiviral constructs expressing Cdc42-specific (shE7 or shE6) or control (shLuc) shRNAs. (K) Multiplex RT-PCR analysis showing that Cdc42-specific shRNAs introduced in (J) alter fractional abundance of the corresponding Cdc42 isoforms. (L) Primary hippocampal neurons were transduced with the constructs in (J) at DIV0 and immunostained for SMI312 at DIV3, and neuronal fractions with zero, one, and more than one positive axon were compared by χ^2^ test. Note significant accumulation of neurons lacking SMI312-positive axons in the shE7 sample as compared to shLuc and shE6. (M) Percentage of neurons in (L) containing no detectable axons was compared by t test. Data in (E), (F), (H), (I), (L), and (M) are from at least three independent litters, with the n values showing overall numbers of neurons analyzed, and in (F), (I), and (M), also the numbers of independent litters. Error bars in (F), (I), and (M) correspond to SE. See also Figure S4.

To examine Cdc42 isoform-specific functions, we altered the Cdc42E6/Cdc42E7 ratio in primary neurons by transducing them with lentiviral vectors producing comparable amounts of corresponding YFP (yellow fluorescent protein)-tagged proteins (YFP-Cdc42E6 or YFP-Cdc42E7; Figures 3A–3C). Consistent with earlier data (Kang et al., 2008), expression of the tagged Cdc42E6 increased the density of post-synaptic puncta as compared to the samples transduced with YFP-Cdc42E7 or YFP empty vector (Figures S4A and S4B).

Cdc42 is also known to promote axon outgrowth (Garvalov et al., 2007, Schwamborn and Püschel, 2004, Toriyama et al., 2013) but it has been unclear whether this function is isoform specific. Strikingly, staining the aforementioned samples for pan-axonal (Tau or SMI312 neurofilament) or axon initial segment (AIS; AnkG) markers showed that YFP-Cdc42E7, but not YFP-Cdc42E6, significantly increased the incidence of neurons containing more than one axon (Figures 3D–3I).

Since the aforementioned strategy likely increased overall Cdc42 expression beyond its physiological level, we used isoform-specific short hairpin RNAs (shRNAs) as an alternative approach (Figures 3J and 3K; Figure S4C). Notably, samples transduced with E7-specific shRNAs (EGFP-shE7) contained significantly elevated fractions of neurons containing no axons, as compared to transductions with E6-specific or control shRNAs (EGFP-shE6 or EGFP-shLuc) (Figures 3L and 3M).

These experiments suggested that the two Cdc42 isoforms might be functionally specialized in neurons with the ubiquitously expressed Cdc42E7 promoting axonogenesis and the neuron-restricted Cdc42E6 stimulating dendritic spine formation.

### Mouse E6-Knockout Model Confirms Functional Specialization of the Two Cdc42 Isoforms

**Figure 4:**
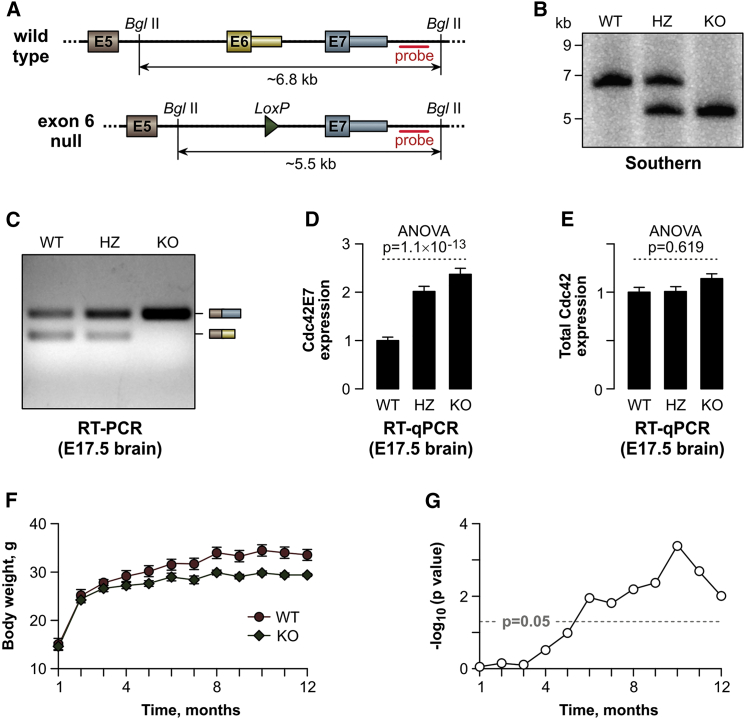
Generation of KO Mice Lacking Cdc42 E6 Sequence (A) Genomic structure of the WT and the E6 null *Cdc42* alleles. Red lines indicate the probe used for Southern blotting. (B) Southern blot of BglII-digested genomic DNA detecting a WT allele-specific ∼6.8-kb product in the WT mice and an E6 null-specific ∼5.5-kb product in the KO animals. Both fragments are present in the heterozygotes (HZ). (C) Multiplex RT-PCR analysis of E17.5 brains, confirming the expected lack of the Cdc42E6 isoform in the KO sample. (D and E) In (D), qRT-PCR with Cdc42E7-specific primers (Cdc42-F1/Cdc42-R2; Table S5) reveals a significant increase in the abundance of this isoform in HZ and KO E17.5 brains upon E6 deletion. (E) qRT-PCR with primers against a constitutively spliced Cdc42 region (Cdc42-F3/Cdc42-R4; Table S5), showing that the overall Cdc42 mRNA levels remain virtually unchanged across the three brain samples. Data are averaged from at least three biological replicates for each genotype ± SD and compared using one-way ANOVA. Cdc42 expression in the WT samples is set to 1. (F) Time-resolved comparison between WT (n = 6) and KO (n = 6) male littermates, showing reduced weight gain in the KO cohort as compared to the WT. Data points are averages ± SE. (G) Log_10_-transformed p values from a two-tailed t test demonstrating that the weight difference between the WT and the KO groups reaches significance by 6 months of age. See also Figure S5 and Table S5.

To test whether functions of the two Cdc42 variants also differed in vivo, we engineered a mouse allele lacking the entire E6 sequence (Figures 4A and 4B). This modification was expected to completely eliminate Cdc42E6 and concomitantly upregulate Cdc42E7 production in neurons. Indeed, the Cdc42E6 mRNA and protein were detectable in embryonic brains from WT (*Cdc42*^*wt/wt*^) and heterozygous (*Cdc42*^*wt/tm1.2Mkv*^; HZ) mice but not from E6 null (*Cdc42*^*tm1.2Mkv/tm1.2Mkv*^; knockout [KO]) mice (Figure 4C; Figures S5A and S5B). Moreover, Cdc42E7 mRNA levels increased progressively from WT to HZ to KO (Figure 4D; Figure S5B), with no detectable change in the total Cdc42 expression (Figure 4E).

**Figure 5:**
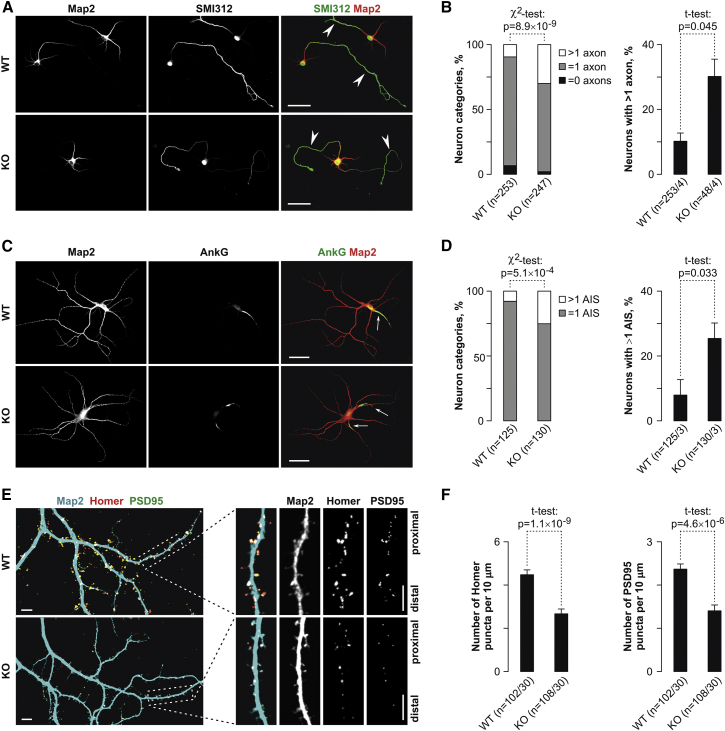
Deregulation of Axo-dendritic Polarity in E6 KO Neurons (A and B) Staining of DIV3 WT and KO hippocampal neurons for axonal (SMI312) and dendritic (Map2) markers reveals a significantly increased incidence of supernumerary axons in the KO. (A) Representative confocal images. (B) Quantitative comparisons between WT and KO carried out as explained in Figures 3E and 3F. (C and D) DIV14 hippocampal neurons immunostained for Map2 and the AIS-specific marker AnkG confirms the supernumerary axon phenotype in the KO neurons. (C) Representative images. (D) Quantitations. (E and F) Staining for Map2, Homer, and PSD95 shows a significantly reduced density of dendritic spines in the KO DIV21 hippocampal neurons as compared to the WT. (E) Representative images with magnified dendritic segments. (F) t test comparisons of WT and KO dendritic spine densities deduced from Homer- and PSD95-specific signals. Scale bars, 50 μm in (A) and (C) and 5 μm in (E). Quantitations in (B), (D), and (F) were carried out using neurons derived from at least three independent litters with the n values showing (B) and (D) numbers of neurons and litters and (F) numbers of neurons and dendritic segments analyzed. Error bars in (B), (D), and (F) correspond to SE. See also Figures S6 and S7.

The KO mice were viable and showed no gross morphological defects (Figures S5C and S5D), suggesting that Cdc42E7 was sufficient for correct brain patterning and neurogenesis in vivo. However, weight gain in KO animals was reduced significantly, compared to that of their WT littermates (Figures 4F and 4G). To begin dissecting mechanisms underlying this phenotype, we stained KO and WT neurons for appropriate axonal and dendritic markers (Figure 5; Figures S6A–S6D). Consistent with Figure 3 and Figure S4, KO neurons showed a significantly increased incidence of supernumerary axons (Figures 5A–5D; Figures S6A−S6D) and a decreased density of post-synaptic puncta compared to the WT (Figures 5E and 5F).

**Figure 6:**
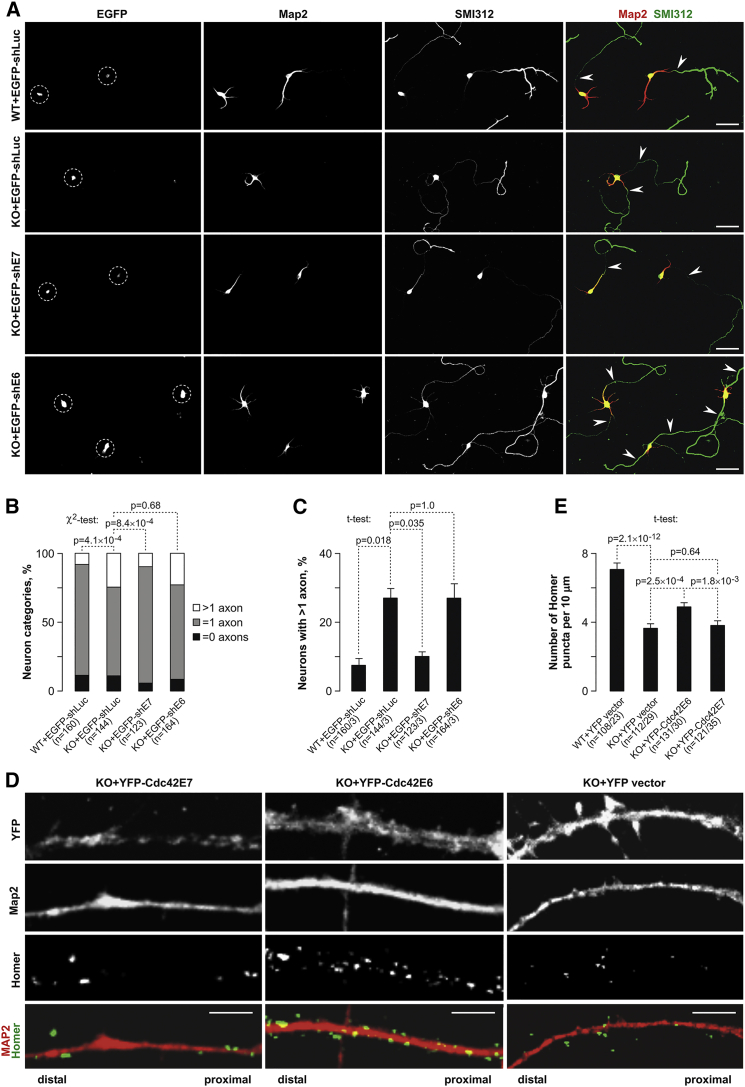
Excessive Axonogenesis in KO Neurons Is due to Cdc42E7 Upregulation, while Reduced Dendritic Spine Density Is a Result of Cdc42E6 Loss (A–C) Increased expression of Cdc42E7 in KO hippocampal neurons was countered by transducing them with the EGFP-shE7 lentivirus (KO+EGFP-shE7) at DIV0, and the neurons were immunostained for SMI312 and Map2 at DIV3. WT+EGFP-shLuc, KO+EGFP-shLuc, and KO+EGFP-shE6 samples were used as controls. (A) Representative images with EGFP-positive somas marked by dashed circles. (B and C) Pairwise comparisons carried out as explained in Figures 3E and 3F, showing that EGFP-shE7, but not EGFP-shLuc or EGFP-shE6, reduces the percentage of KO neurons containing more than one axon to a WT-like level. (D and E) Loss of the Cdc42E6 expression was rescued by transducing KO hippocampal neurons with the YFP-Cdc42E6 at DIV0 followed by immunostaining with Map2- and Homer-specific antibodies at DIV21. YFP vector and YFP-Cdc42E7 constructs were used as controls. (D) Representative dendritic segments of transduced neurons corresponding to lower magnification images in Figure S6H. (E) t test comparisons of dendritic spine densities for WT and KO neurons transduced with corresponding expression constructs showing that YFP-Cdc42E6 at least partially restores spines lost in the absence of endogenous Cdc42E6. Scale bars, 100 μm in (A) and 5 μm in (D). Data in (B), (C), and (E) are averaged from at least three independent experiments, with the error bars representing SE. Quantitations in (B), (C), and (E) were done using neurons from at least three independent litters with the n values showing (B) total numbers of neurons and (C and E) both numbers of neurons and litters analyzed. Error bars in (C) and (E) correspond to SE. See also Figures S6 and S7.

Importantly, dampening elevated Cdc42E7 expression in KO neurons with shE7 brought the number of axons back to normal (Figures 6A–6C) while having no significant effect on the dendrites (Figures S6E and S6F). Control shRNAs (shLuc and shE6; Figures 6A–6C) and YFP-Cdc42E6 overexpression (Figure S6G) failed to rescue this phenotype. On the other hand, the decrease in the dendritic spine density was partially reversed when we transduced KO neurons with YFP-Cdc42E6 but not YFP-Cdc42E7 or YFP-vector constructs (Figures 6D and 6E; Figure S6H).

To ensure that the effects of the E6 deletion observed in primary neuronal cultures were also present in mouse brain, we examined the morphology of pyramidal neurons in sparsely labeled hippocampal slices (Figure S7). Compared to the WT, KO neurons showed significantly reduced density of dendritic spines on arbors containing these structures (Figures S7A and S7B), as well as significantly increased incidence of axon-like projections completely devoid of dendritic spines (Figures S7A and S7C). At least in some EGFP-labeled neurons, AIS structures could be discerned in these axon-like projections by immunostaining for AnkG (Figure S7D).

We concluded that Cdc42E6 and Cdc42E7 are functionally specialized in neurons and that their balanced co-expression is essential for proper development of the dendritic and the axonal compartments, respectively.

## Discussion

AS is commonly thought to elaborate organismal gene expression through generating tissue- and developmental-stage-specific products (Kalsotra and Cooper, 2011, Maniatis and Tasic, 2002, Nilsen and Graveley, 2010, Pan et al., 2008, Wang et al., 2008). Our study suggests that AS may additionally provide a widespread mechanism for increasing the number of functionally distinct protein isoforms within individual cells. Using A3E regulation during mammalian neurogenesis as a model system, this work uncovers a number of cases where splice isoforms are persistently co-expressed in terminally differentiated neurons.

**Figure 7:**
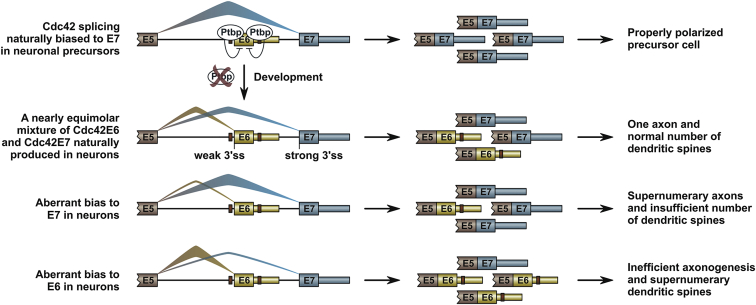
Model Outlining Functional Significance of Regulated Cdc42 Splicing in Developing Neurons Non-neuronal cells express relatively large amounts of Ptbp1, inhibiting E6 and biasing Cdc42 splicing towards E7 inclusion. Ptbp1 down-regulation in neurons promotes sustained co-expression of functionally specialized Cdc42E6 and Cdc42E7 isoforms. Deregulation of the natural balance between these two isoforms leads to defects in the axonal and the dendritic compartments. See text for more detail.

In a representative example of this category, co-expression of the two Cdc42 isoforms is orchestrated by a combination of Ptb protein-dependent and -independent mechanisms (Figure 7). Evocative of several AS events described earlier (Keppetipola et al., 2012, Llorian et al., 2010, Yap et al., 2012), the E6 exon is repressed early in development, in part, through recruitment of Ptbp1 and/or Ptbp2 to ePE and iPE clusters of Ptbp1/2 consensus binding motifs (Figure 2A; Figure S3A). Therefore, miR-124-mediated downregulation of Ptbp1 at early stages of neurogenesis (Makeyev et al., 2007) and subsequent decline in the Ptbp2 levels in mature neurons (Zheng et al., 2012) are expected to stimulate E6 inclusion in vivo. Our in situ hybridization data are consistent with the role of Ptbp1 in regulating the E7/E6 switch in the developing neural tube (Figure 2E). Of note, mutation of the ePE and iPE sequences significantly reduced, but did not completely eliminate, the Ptbp1/2 dependence of E6 (Figures 2C and 2D). This residual regulation may rely on the remaining nine Ptbp1/2 motifs at the intron 5-E6 junction (Figure S3A).

Besides its role in splicing regulation, Ptbp1 has been reported to modulate pre-mRNA cleavage and polyadenylation for several 3′-terminal exons by interacting with adjacent pyrimidine-rich sequences (Castelo-Branco et al., 2004, Le Sommer et al., 2005). However, since the 3′ terminus of E6 is dispensable for the regulation (Figures 2A and 2B), Ptb proteins likely repress this exon by diminishing the efficiency of its splicing acceptor rather than inhibiting cleavage/polyadenylation.

Notably, the switch from E7 to E6 upon Ptbp1/2 inactivation remains incomplete, despite the more favorable proximal position of E6 in the Cdc42 pre-mRNA (Figure 2). This may explain the lasting co-expression of the two isoforms in neurons detected by RT-PCR (Figures 1C and 1D; Figure S2A), RNA FISH (Figure 1E), and qRT-PCR analyses (Figure S5B). We show that this behavior depends on the splicing acceptor sequence of E7 being substantially stronger than its E6 counterpart (Figures S3I and S3J) and hypothesize that the relative strengths of these sequences might have been evolutionarily optimized to deliver a nearly equimolar blend of the two isoforms in neurons. Co-expression of the E6- and E7-terminated products may also point to a considerable delay between transcription and splicing of this part of the Cdc42 pre-mRNA, which would be consistent with earlier transcriptome analyses (Ameur et al., 2011, Girard et al., 2012).

Importantly, we show that the Cdc42E7 isoform has its own function in developing neurons, where it stimulates axon specification in a dose-dependent manner (Figures 3, 5, 6, and S7). Our data (Figures 5, 6, S4, and S7) also support the previously proposed role of Cdc42E6 in regulating dendritic spine morphogenesis (Kang et al., 2008, Wirth et al., 2013). This segregation of duties between co-expressed Cdc42E6 and Cdc42E7 in neurons would explain the broad range of activities reported for Cdc42 in this cell type (Cappello et al., 2006, Chen et al., 2006, Chen et al., 2012, Garvalov et al., 2007, Kang et al., 2008, Mukai et al., 2015, Schwamborn and Püschel, 2004).

The relatively mild phenotypic consequences of deleting E6 in mouse are in line with upregulation of this isoform relatively late in neurogenesis, following differentiation of precursor cells neuronal precursors into neurons (Figure 1C; Figure S2A). It is also possible that functions of Cdc42E7 and Cdc42E6 are partially interchangeable outside of the hippocampus. This would be consistent with a recent study implicating Cdc42E6 as a regulator of axonal length and branching properties of cortical neurons (Mukai et al., 2015). Further work will likely uncover additional phenotypic differences between Cdc42E6 null mice and their WT littermates. For example, our preliminary analyses indicate that KO animals might suffer from increased anxiety (K.Y. and E.V.M., unpublished data), and it will be interesting to see whether this behavioral defect is linked with the reduced weight phenotype (Figures 4F and 4G).

Although additional work will be required to gain mechanistic insights into functional specialization of Cdc42E6 and Cdc42E7, it is logical to assume that it relies on differential cellular localization of the two protein products. Indeed, distinct C-terminal lipid modifications have been shown to result in preferential localization of Cdc42E6, but not Cdc42E7, to dendritic spines (Kang et al., 2008). Moreover, marked differences between the E6 and E7 3′-UTRs might potentially restrict protein isoform production to corresponding neuronal compartments through localized mRNA translation (Andreassi and Riccio, 2009, Jung et al., 2012).

Besides its effect on the proteome composition, AS is known to regulate expression outputs of many genes by modulating mRNA stability, localization, and translational efficiency (Braunschweig et al., 2013, Yap and Makeyev, 2013, Zheng and Black, 2013). The E6/E7 regulation described here provides a notable example where AS simultaneously generates functionally distinct protein products and carefully balances their relative expression levels (Figure 7). It will be interesting to see whether similar logic applies to other important Ptbp1/2-regulated genes, including Cdc42 GEF intersectin 1, G protein alpha subunit Gnas, septin Sept11, and neuronal adhesion molecule Ncam1.

In conclusion, our study suggests that AS may substantially increase the functional complexity of the neuronal proteome at a single-cell level. Arguing for biological importance of this mechanism, carefully controlled balance between two Cdc42 isoforms in neurons ensures the development of a single axon and a normal complement of dendritic spines. We predict that extending quantitative analyses of isoform co-expression to other categories of alternative exons and elucidating functional differences between co-expressed isoforms will substantially advance our understanding of the part played by AS in the development and evolution of eukaryotic organisms.

## Experimental Procedures

### UA3E Expression Analyses

Annotated UA3Es were extracted from the UCSC Known Genes dataset (http://genome.ucsc.edu/) using ExpressionPlot utility scripts (Friedman and Maniatis, 2011) and further filtered to remove other types of regulated RNA processing, e.g., cassette exons or alternative polyadenylation. Mouse glutamatergic neurogenesis RNA-seq data series (Hubbard et al., 2013) contained three to five replicates of each of the DIV minus 8, DIV minus 4, DIV0, DIV1, DIV7, DIV16, DIV21, and DIV28 time points corresponding to ESCs, NSCs, RGCs and five stages of glutamatergic neuron development (GN1–GN5).

### Statistical Analyses

Unless indicated otherwise, experiments were carried out in triplicate, and samples were compared using Student’s two-tailed t test, assuming unequal variances, or one-way ANOVA. See Supplemental Information for a description of other experimental and statistical procedures.

## Author Contributions

K.Y. and E.V.M. conceived the study. H.S.J. and E.V.M. secured funding. K.Y., Y.X., H.S.J., and E.V.M. conducted experiments and analyzed results. B.A.F. and E.V.M. performed bioinformatics analyses. E.V.M. wrote the manuscript, with some help from other authors.

## Supplemental Information

Document S1. Supplemental Experimental Procedures, Figures S1–S7, and Tables S2–S6

Table S1. Enrichment of RBP Motifs in the 426 Regulated UA3Es Compared to the 769 Non-regulated Ones Highly enriched motifs are shaded in blue. See Supplemental Experimental Procedures for more detail.

Document S2. Article plus Supplemental Information
