# Supplementary material for: Polarizing the Neuron through Sustained Co-expression of Alternatively Spliced Isoforms
Source: Cell Rep. 2016 Apr 28;15(6):1316–28. doi: 10.1016/j.celrep.2016.04.012 (PMC4870516; doi:10.1016/j.celrep.2016.04.012)
Supplement: Document S1. Supplemental Experimental Procedures, Figures S1–S7, and Tables S2–S6 [file mmc1.pdf]

Cell Reports, Volume *15*

Supplemental Information

Polarizing the Neuron through Sustained Co-expression of Alternatively Spliced Isoforms

Karen Yap, Yixin Xiao, Brad A. Friedman, H. Shawn Je, and Eugene V. Makeyev

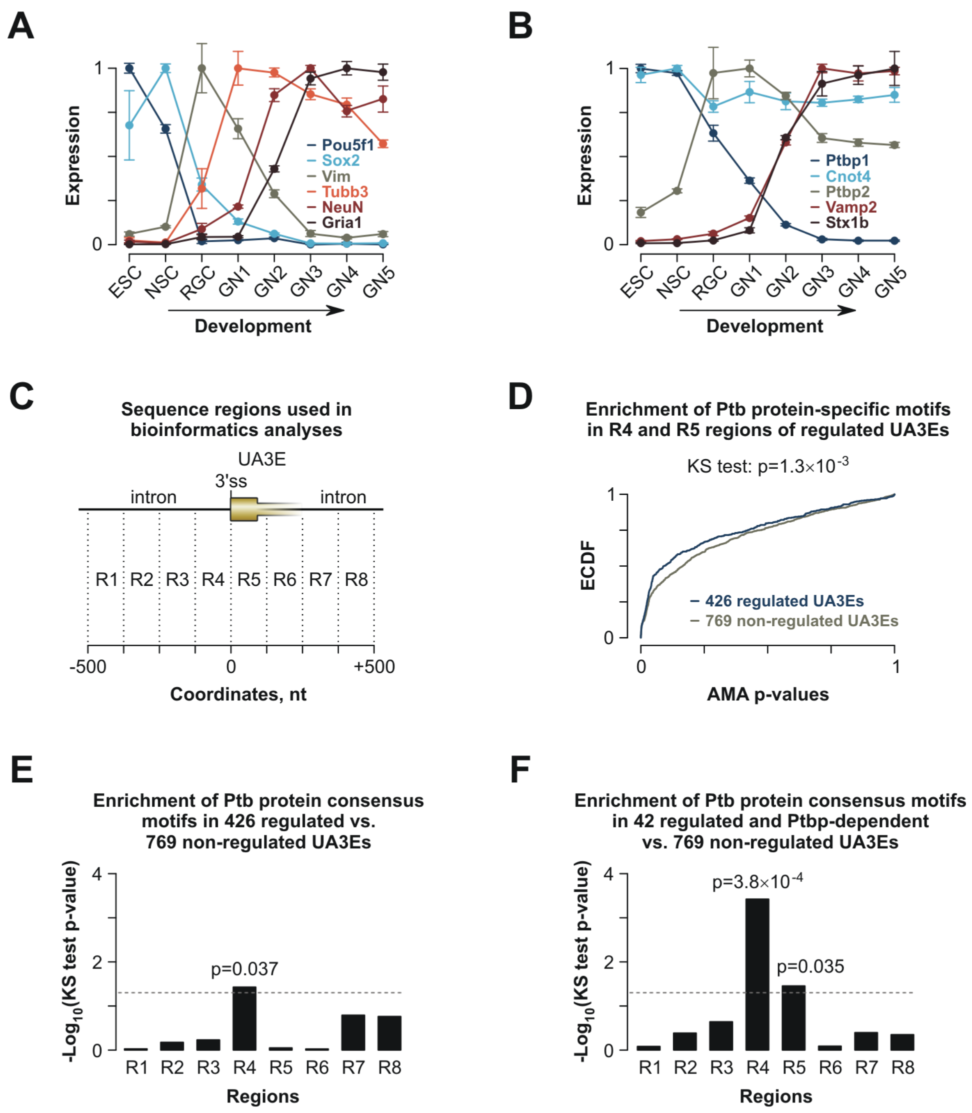

Figure S1

Figure S1. Gene expression and UA3E splicing changes in developing neurons. Related to Figure 1.
(**A-B**) Normalized expression levels of (A) stage-specific neurogenesis markers (Corbin et al., 2008; Jerabek et al., 2014; Menezes and Luskin, 1994; Mullen et al., 1992; Orlandi et al., 2011; Pevny and Nicolis, 2010) and (B) Ptbp1, Ptbp2 and two Ptbp1/2-repressed targets, Stx1b and Vamp2, in ESCs undergoing in vitro differentiation into glutamatergic neurons. Consistent with previous reports (Makeyev et al., 2007; Yap et al., 2012; Zheng et al., 2012), Ptbp1 is developmentally down-regulated, Ptbp2 is transiently up-regulated followed by a detectable decline later in neuronal development, and Stx1b and Vamp2 reach maximal levels in mature neurons. Note that expression of the Cnot4 mRNA control encoding a subunit of the ubiquitous Ccr4-Not complex (Miller and Reese, 2012) remains virtually unchanged. Expression values were calculated using ExpressionPlot (Friedman and Maniatis, 2011) analysis of the corresponding RNA-seq data series (Hubbard et al., 2013).

(**C**) Eight sequence regions (R1-R8) adjacent to the UA3E 3'ss were considered in the motif enrichment analyses in (D-E) and Table S1.
(**D**) Ptb family-specific motifs defined in the CisBP-RNA database as a position weight matrix were analyzed in the regions immediately preceding and following the 3'ss [combined R4 and R5 defined in (C)] using the average motif affinity (AMA) procedure (Buske et al., 2010). AMA p-values for the 426 regulated UA3Es were significantly lower than for the 769 non-regulated UA3Es ($p=1.3\times10^{-3}$; one-sided Kolmogorov-Smirnov (KS) test).
(**E**) As an alternative approach, we checked if Ptbp1/2 consensus binding sequences (YUCUUY, YUCUCY, YUUCUY and YCUCUY) were enriched in the 426 regulated vs. the 769 non-regulated UA3Es. Of the 8 regions defined in (C), significant enrichment was detected in R4 ($p=0.037$; one-sided KS test).
(**F**) Repeating the analysis in (E) for 42 Ptbp1/2-dependent UA3Es vs. the 769 non-regulated ones revealed a striking enrichment of the consensus sequences in R4 ($p=3.8\times10^{-4}$; one-sided KS test) as well as some enrichment in R5 ($p=0.035$). Dashed lines in (E-F) correspond to $p=0.05$.

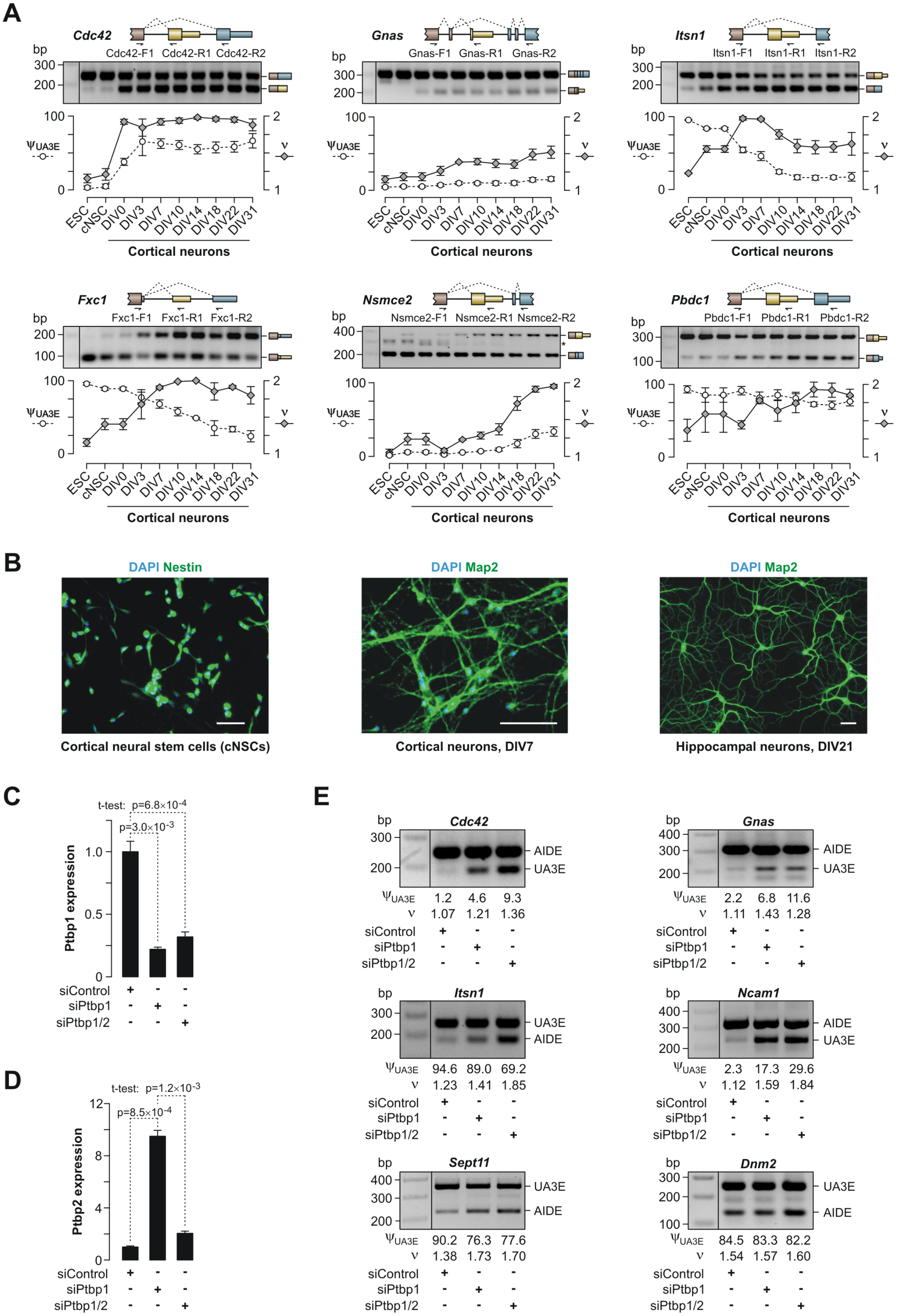
A
Cdc42
Cdc42-F1 Cdc42-R1 Cdc42-R2
Gnas
Gnas-F1 Gnas-R1 Gnas-R2
Itsn1
Itsn1-F1 Itsn1-R1 Itsn1-R2
Fxc1
Fxc1-F1 Fxc1-R1 Fxc1-R2
Nsmce2
Nsmce2-F1 Nsmce2-R1 Nsmce2-R2
Pbdc1
Pbdc1-F1 Pbdc1-R1 Pbdc1-R2
ΨUA3E
ν
ESC cNSC DIV0 DIV3 DIV7 DIV10 DIV14 DIV18 DIV22 DIV31
Cortical neurons
B
DAPI Nestin
DAPI Map2
Cortical neural stem cells (cNSCs)
Cortical neurons, DIV7
Hippocampal neurons, DIV21
C
t-test: p=6.8×10-4
p=3.0×10-3
Ptbp1 expression
siControl
siPtbp1
siPtbp1/2
D
t-test: p=1.2×10-3
p=8.5×10-4
Ptbp2 expression
E
Cdc42
AIDE
UA3E
ΨUA3E 1.2 4.6 9.3
ν 1.07 1.21 1.36
Gnas
ΨUA3E 2.2 6.8 11.6
ν 1.11 1.43 1.28
Itsn1
ΨUA3E 94.6 89.0 69.2
ν 1.23 1.41 1.85
Ncam1
ΨUA3E 2.3 17.3 29.6
ν 1.12 1.59 1.84
Sept11
ΨUA3E 90.2 76.3 77.6
ν 1.38 1.73 1.70
Dnm2
ΨUA3E 84.5 83.3 82.2
ν 1.54 1.57 1.60

Figure S2

Figure S2. Many UA3E/AIDE pairs are persistently co-expressed in primary neurons and are regulated by Ptbp1/2. Related to Figures 1 and 2.

(**A**) Ptbp1/2-dependent (Cdc42, Gnas and Itsn1) and -independent (Fxc1, Nsmce2 and Pbdc1) UA3E/AIDE pairs with increasing co-expression trend were analyzed by multiplex RT-PCR in mouse ESCs, cortical NSCs (cNSCs) and primary cortical neurons at different stages of maturation (DIV0-DIV31). Relevant gene fragments and PCR primers used for the analysis are shown on the top and $\Delta\psi_{UA3E}$ and ν time-course analyses are provided at the bottom of each gel image. Data are averaged from 3 experiments using independent cell cultures ±SE.

(**B**) Immunofluorescence analyses confirming homogeneity of cNSCs and the two types of primary neurons used in our experiments. Note that all cells co-stain with a nuclear dye (DAPI) and corresponding cell type-specific markers: nestin (NSCs) or Map2 (neurons). Scale bar, 50 µm.

(**C-D**) CAD cells were transfected with siControl, siPtbp1 and siPtbp1/2 and analyzed by RT-qPCR 72 hours post-transfection for the effects of these siRNAs on (A) Ptbp1 and (B) Ptbp2 expression levels. Note that both siPtbp1 and siPtbp1/2 efficiently knock down Ptbp1. siPtbp1 also dramatically increases Ptbp2 levels, which is expected given that Ptbp1 protein represses Ptbp2 mRNA expression through AS coupled with nonsense-mediated decay (Boutz et al., 2007; Makeyev et al., 2007; Spellman et al., 2007). This up-regulation effect is largely mitigated in the siPtbp1/2 samples.

(**E**) Splicing patterns of 6 predicted Ptb protein-dependent UA3E/AIDE pairs were analyzed in the above siControl, siPtbp1 and siPtbp1/2 samples using multiplex RT-PCR with appropriate gene-specific F1/R1/R2 primer mixtures (Table S5). Combined knockdown of both Ptbp1 and Ptbp2 tends to trigger a more pronounced AS switch than knockdown of Ptbp1 for all genes except *Sept11*. Also note that a reduction in the Ptb protein levels leads to an increase in the isoform co-expression index ν.

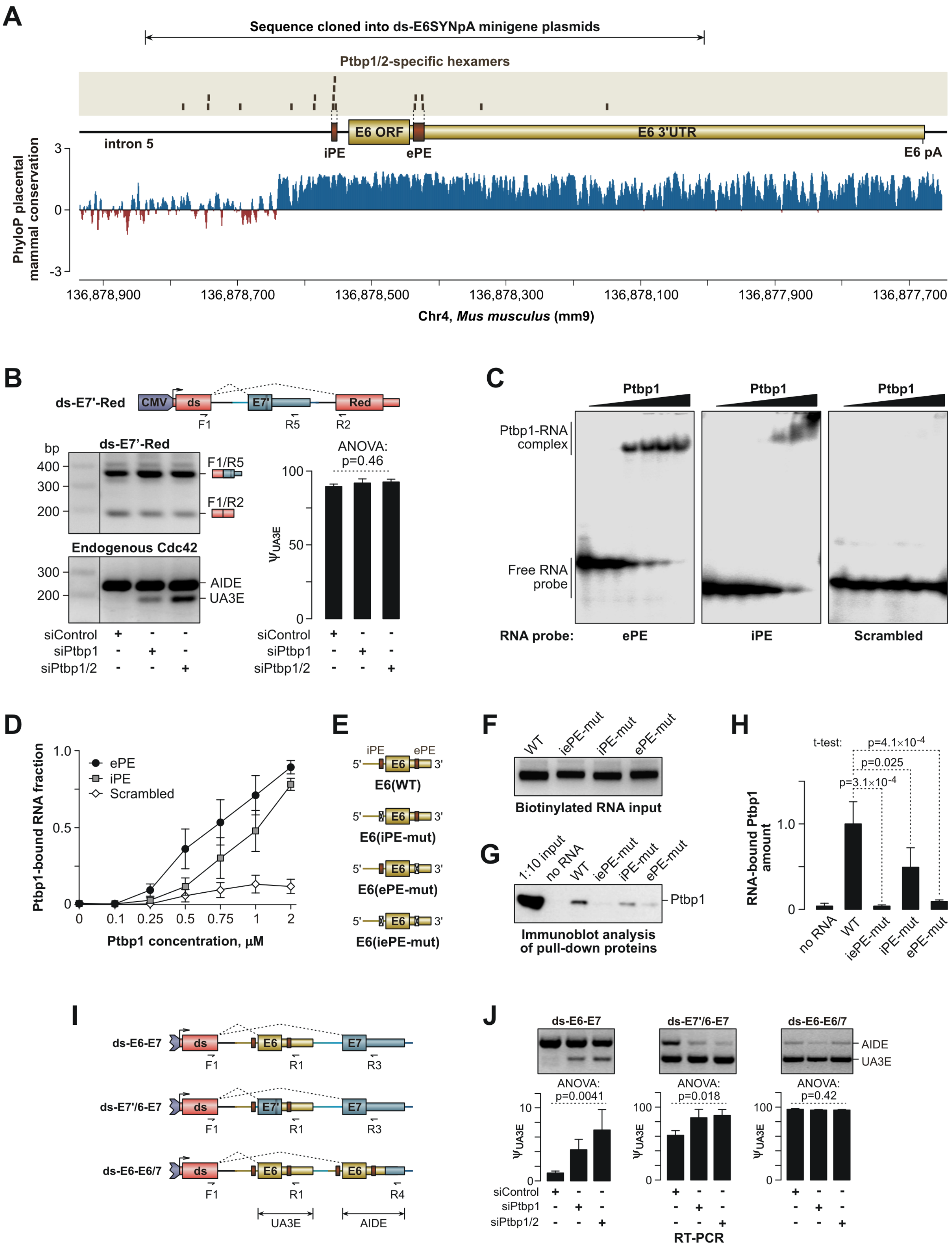

A
Sequence cloned into ds-E6SYNpA minigene plasmids
Ptbp1/2-specific hexamers
intron 5
E6 ORF
E6 3'UTR
iPE
ePE
E6 pA
PhyloP placental mammal conservation
Chr4, Mus musculus (mm9)
B
ds-E7'-Red
CMV
ds
E7'
Red
F1
R5
R2
Endogenous Cdc42
AIDE
UA3E
ANOVA: p=0.46
siControl
siPtbp1
siPtbp1/2
C
Ptbp1
Ptbp1-RNA complex
Free RNA probe
RNA probe:
ePE
iPE
Scrambled
D
ePE
iPE
Scrambled
Ptbp1-bound RNA fraction
Ptbp1 concentration, μM
E
E6(WT)
E6(iPE-mut)
E6(ePE-mut)
E6(iePE-mut)
F
WT
iePE-mut
iPE-mut
ePE-mut
Biotinylated RNA input
G
1:10 input
no RNA
Ptbp1
Immunoblot analysis of pull-down proteins
H
t-test:
p=4.1×10-4
p=0.025
p=3.1×10-4
RNA-bound Ptbp1 amount
I
ds-E6-E7
ds-E7'/6-E7
ds-E6-E6/7
F1
R1
R3
R4
UA3E
AIDE
J
ANOVA: p=0.0041
ANOVA: p=0.018
ANOVA: p=0.42
RT-PCR

Figure S3

Figure S3. Molecular mechanisms regulating splicing choice between E6 and E7 exons in Cdc42 pre-mRNA. Related to Figure 2.

(**A**) Distribution of 18 Ptbp1/2-specific pyrimidine-rich motifs (YTCTTY, YTCTCY, YTTCTY and YCTCTY) in the vicinity of Cdc42 E6. Sequence fragment cloned into ds-E6SYNpA-Red minigene constructs is indicated at the top and base-wise phyloP conservation (Pollard et al., 2010) of Cdc42 sequences across placental mammals is shown at the bottom.

(**B**) Utilization of E7 does not depend on Ptb proteins. *Top*, ds-E7'-Red minigene containing a modified version of Cdc42 exon 7 within its natural genomic context. An internal sequence of the wild-type E7 (CAGGTGTGTGCT) can be aberrantly recognized as a splicing donor when this exon is moved to an UA3E position in a minigene context. To avoid this undesirable effect, we mutated this sequence in E7' to (CAGCACACAGCT). *Left*, multiplex RT-PCR showing that siPtbp1 and siPtbp1/2 have no effect on E7' inclusion while regulating endogenous Cdc42 splicing. *Right*, quantitation of the ds-E7'-Red splicing data confirming that Ptbp1 and Ptbp2 have no significant effect on exon 7 splicing. Data are averaged from 3 experiments ±SD and analyzed by one-way ANOVA.

(**C-H**) Ptbp1 directly interacts with Cdc42 exon 6 splicing acceptor-proximal sequences. (C) Electromobility shift assays (EMSAs) showing that purified recombinant Ptbp1 protein interacts with exonic and intronic pyrimidine-rich sequences (ePE and iPE, respectively) but not with a "scrambled" sequence. Ptbp1 concentrations were increased from 0 to 2 μM. (D) Quantification of the data in (C) suggesting that ePE has a relatively higher Ptbp1-binding affinity than iPE. Data are averaged from 3 independent EMSA experiments ±SE. (E) Biotinylated RNA baits comprising either wild-type or mutated versions of iPE and ePE in their natural Cdc42 exon 6 splicing acceptor context. (F) Equal amounts (1 μg each) of the RNA baits introduced in (E) analyzed by agarose gel electrophoresis. (G) Immunoblot analysis of Ptbp1 protein pulled down from HeLa nuclear extract by indicated RNA baits. (H) Quantification of Ptbp1 signal intensity in (G). Note that the wild-type bait readily binds Ptbp1 and this interaction is diminished by mutating PEs individually (iPE-mut or ePE-mut) and completely abolished by the double mutation (iePE-mut). Data are averaged from 4 independent pull-down experiments ±SD and compared by two-tailed t-test.

(**I**) Minigenes containing either wild-type or modified Cdc42 UA3E and AIDE. Arrows indicate primers used for multiplex RT-PCR.

(**J**) Multiplex RT-PCR analyses of splicing patterns for the minigenes in (I) suggests that the AS switch in Ptbp1/2-depleted cells is incomplete because Cdc42 E7 has a constitutively stronger 3'ss than E6. *Top*, RT-PCR products separated by agarose gel electrophoresis. *Bottom*, UA3E-specific percent-spliced-in values (ψ_{UA3E}). Data are averaged from three independent experiments ± SD and compared using one-way ANOVA.

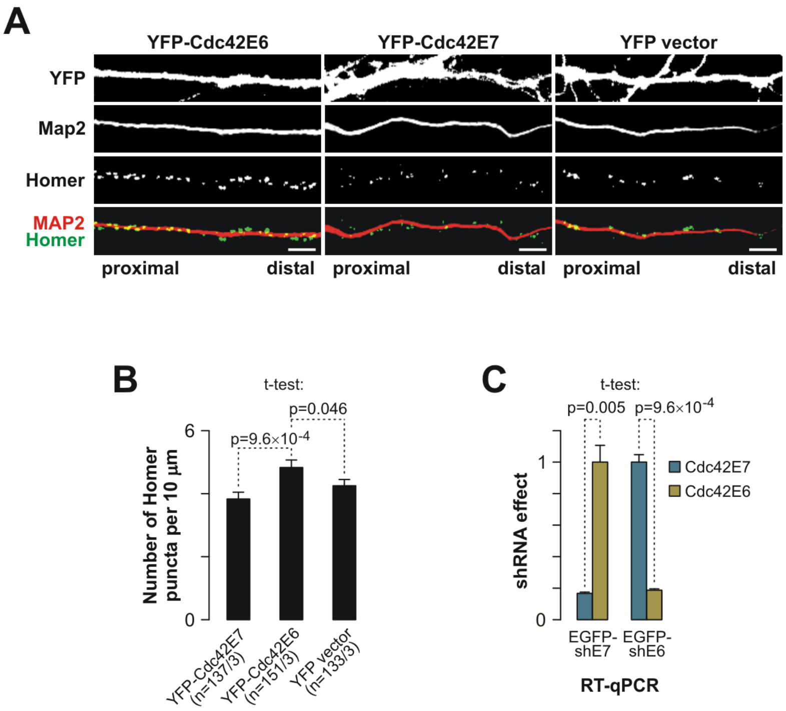

Figure S4

Figure S4. Overexpression and knockdown of Cdc42E6 and Cdc42E7 isoforms in primary neurons. Related to Figure 3.

(**A**) Representative arbors of primary hippocampal neurons transduced with YFP-Cdc42E6, YFP-Cdc42E7 or YFP vector constructs at DIV0 and immunostained for the dendritic marker Map2 and the dendritic spine marker Homer at DIV21. Scale bars, 5 µm.

(**B**) Quantitation of the images in (A) showing a significantly higher density of Homer-positive puncta in YFP-Cdc42E6 samples compared to YFP vector or YFP-Cdc42E7. Data are from 3 independent experiments with the *n* values indicating numbers of dendritic segments used for quantitation and the total numbers of neurons.

(**C**) Neuronal cultures transduced with shRNA constructs from Fig. 3J at DIV0 and analyzed by RT-qPCR at DIV3 for efficiency of isoform-specific knockdown. Effects of shRNA on non-specific isoforms (i.e. Cdc42E6 for shE7 and Cdc42E7 for shE6) are set to 1. Data are averaged from 3 experiments ±SD.

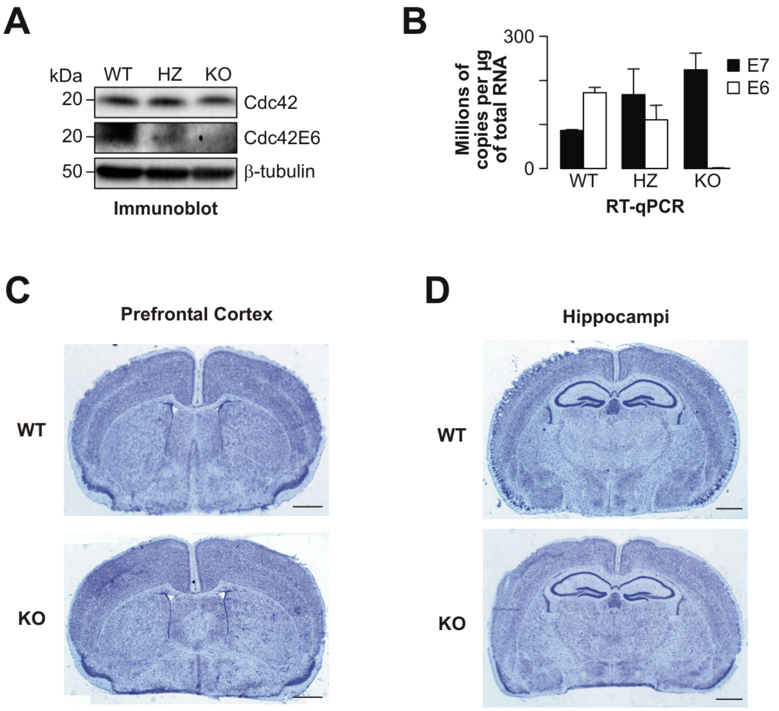

Figure S5

Figure S5. Characterization of Cdc42E6-null KO mice. Related to Figure 4.

(**A**) Immunoblot analyses of WT, HZ and KO brains using general and E6 isoform-specific anti-Cdc42 antibodies confirm the loss of the Cdc42E6 isoform with no change in the overall Cdc42 protein levels in the KO. β-tubulin is used as a lane loading control.

(**B**) Absolute RT-qPCR quantitation of Cdc42E6 and Cdc42E7 mRNA copy numbers per 1 µg of total RNA in E17.5 mouse brain. Calibration curves were generated using in vitro transcribed Cdc42E6 and Cdc42E7 RNA fragments.

(**C-D**) Representative images of Nissl-stained 40 µm-thick coronal sections of WT and KO adult (P21) mouse brains at the level of prefrontal cortex and hippocampus, respectively.

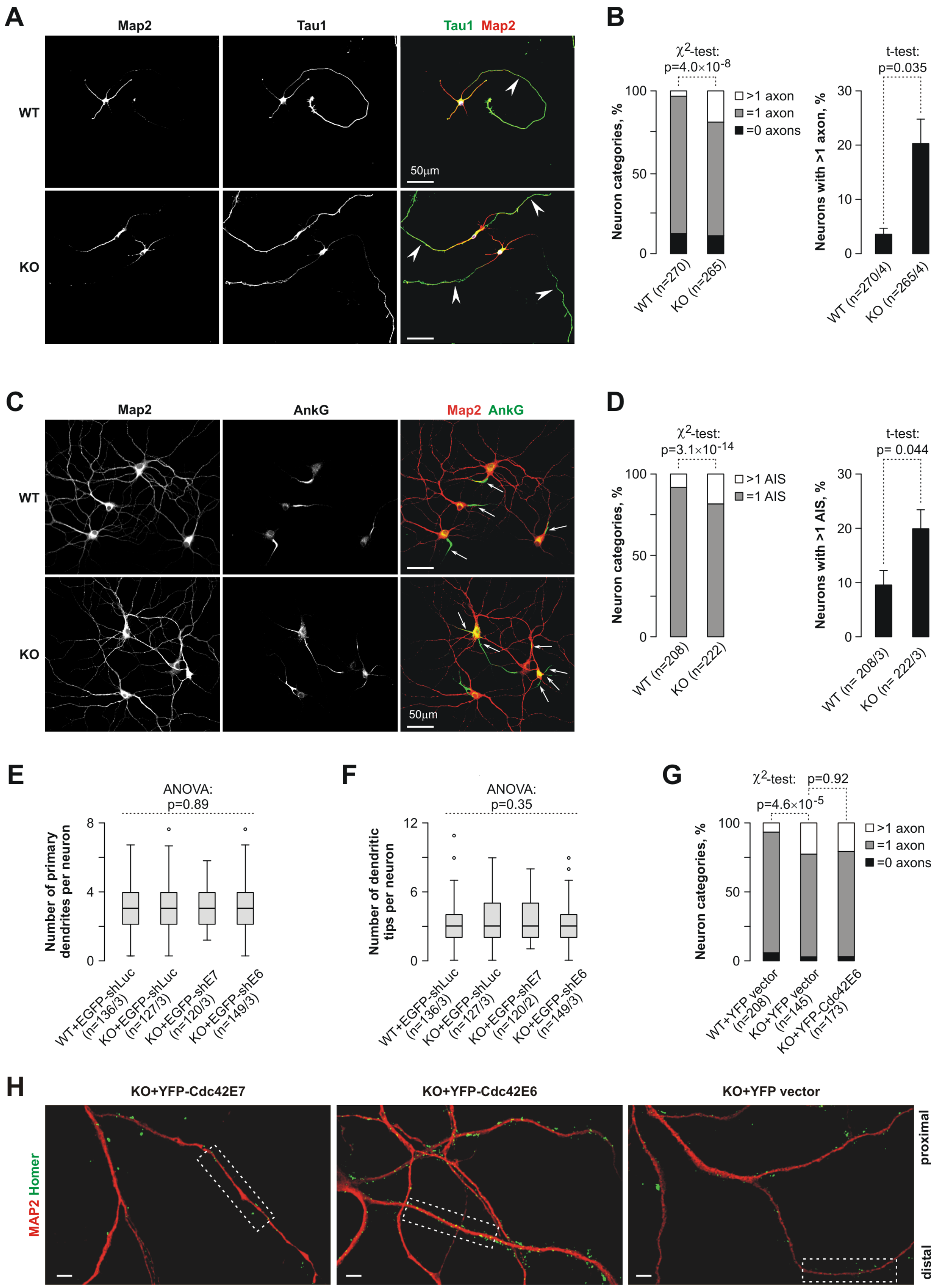
A
Map2
Tau1
Tau1 Map2
WT
KO
50µm
B
χ2-test:
p=4.0×10-8
>1 axon
=1 axon
=0 axons
Neuron categories, %
WT (n=270)
KO (n=265)
t-test:
p=0.035
Neurons with >1 axon, %
WT (n=270/4)
KO (n=265/4)
C
Map2
AnkG
Map2 AnkG
D
χ2-test:
p=3.1×10-14
>1 AIS
=1 AIS
WT (n=208)
KO (n=222)
t-test:
p= 0.044
Neurons with >1 AIS, %
WT (n= 208/3)
KO (n= 222/3)
E
ANOVA:
p=0.89
Number of primary dendrites per neuron
WT+EGFP-shLuc (n=136/3)
KO+EGFP-shLuc (n=127/3)
KO+EGFP-shE7 (n=120/3)
KO+EGFP-shE6 (n=149/3)
F
ANOVA:
p=0.35
Number of dendritic tips per neuron
KO+EGFP-shE7 (n=120/2)
G
χ2-test:
p=4.6×10-5
p=0.92
WT+YFP vector (n=208)
KO+YFP vector (n=145)
KO+YFP-Cdc42E6 (n=173)
H
KO+YFP-Cdc42E7
KO+YFP-Cdc42E6
KO+YFP vector
MAP2 Homer
proximal
distal

Figure S6

Figure S6. Cdc42E6-null KO neurons generate supernumerary axons as a result of Cdc42E7 gain of function. Related to Figures 5 and 6.

(**A, C**) Representative confocal images of WT and KO hippocampal neurons at (A) DIV3 and (C) DIV18 stained with Tau1-, AnkG- and Map2-specific antibodies as indicated. Note that unlike WT, KO neurons often develop >1 Tau-positive axon (arrowheads) or AnkG-positive AIS (arrows). Scale bars, 50 µm.

(**B, D**) Quantification of the data in (A) and (C), respectively, carried out as in Fig. 3E-F. Data are averaged from three independent experiments ±SE and compared using χ^2- or two-tailed t-test. *n* values show total numbers of neurons and, in the case of t-test comparisons, numbers of independent litters analyzed.

(**E-F**) One-way ANOVA box plot comparisons showing no significant difference in numbers of primary dendrites and dendritic tips per neuron among WT and KO samples treated with indicated shRNAs. *n* values indicate total numbers of neurons and independent litters, respectively.

(**G**) Expressing YFP-Cdc42E6 in KO hippocampal neurons fails to rescue the supernumerary axon phenotype. *n* values indicate total numbers of neurons used for the analysis.

(**H**) Lower magnification images corresponding to Fig. 6D showing KO hippocampal neurons transduced with the indicated constructs at DIV0, fixed at DIV21 and labeled with Map2 and Homer antibodies. Note that YFP-Cdc42E6-trasduced neurons have a higher density of Homer-positive puncta than the two other samples. Scale bar, 5 µm.

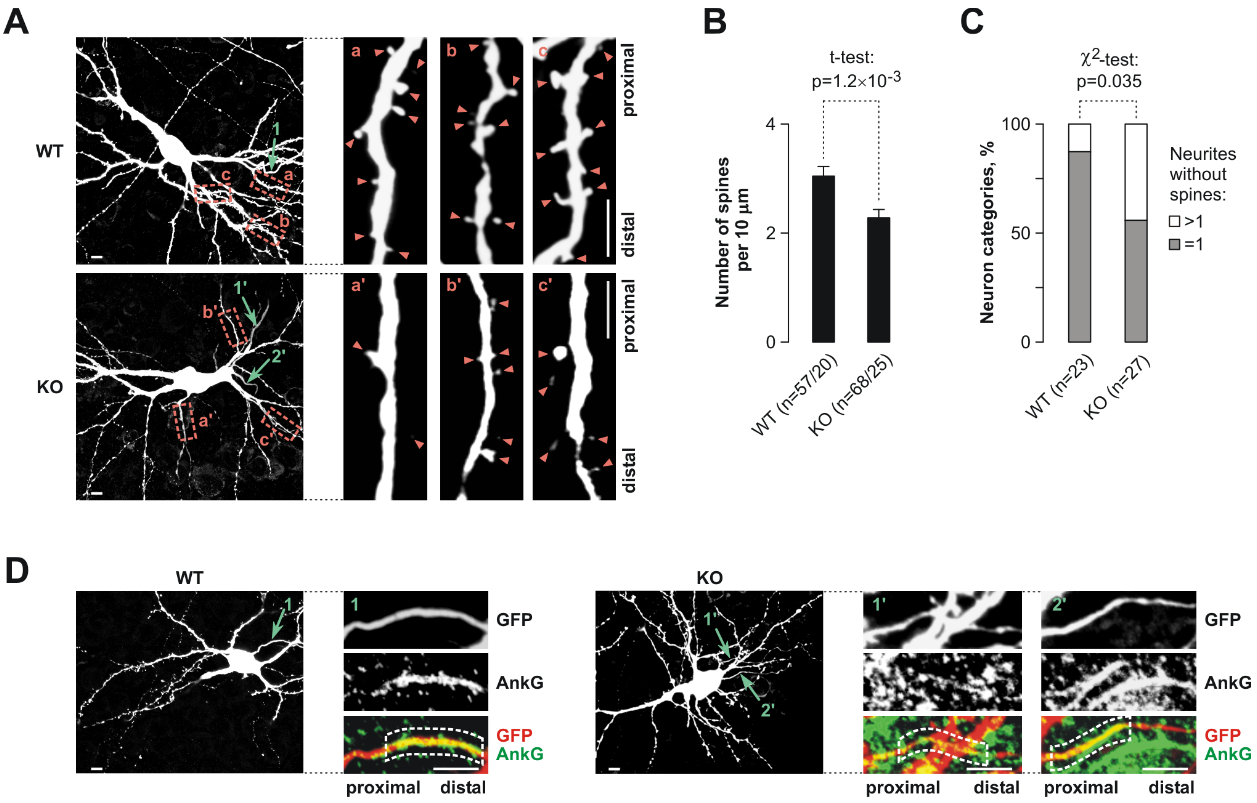

Figure S7

Figure S7. Balanced co-expression of Cdc42 isoforms is required for proper axonal and dendritic development *in vivo*. Related to Figures 5 and 6.

(**A**) Hippocampal pyramidal neurons were labeled with EGFP in postnatal brain slices as described in Supplemental Experimental Procedures and analyzed by confocal microscopy. *Top*, a WT neuron with branched spine-containing dendrites and a single axon characterized by the absence of spines (light green arrow 1). Close-up images on the right provide a better view of spines (pink arrowheads) for randomly selected secondary dendritic branches a, b and c. *Bottom*, a KO neuron containing several spine-containing dendrites as well as two axon-like projections completely devoid of spines (light green arrows 1' and 2'). Close-ups on the right show that secondary dendritic branches (a', b' and c') in the KO have noticeably reduced density of spines (pink arrowheads) compared to the WT control. KO spines also tend to be thinner that the WT ones.

(**B**) t-test comparisons of dendritic spine densities in (A). *n* values show the numbers of dendritic segments and the total numbers of neurons analyzed.

(**C**) χ^2-test comparison of neuronal categories containing 1 or >1 axon-like projections completely devoid of spines in WT and KO hippocampi. *n* are numbers of neurons analyzed.

(**D**) Combined confocal analysis of EGFP and AnkG immunofluorescence confirming axonal identity of spine-less neurites. Low magnification images on the left of the WT and KO image sets show overall neuronal morphology visualized using EGFP fluorescence and the close-ups on the right correspond to AnkG-positive AIS parts (dashed outlines) of axons. Note that the WT neuron has one AIS (light green arrows 1) and the KO has two AISs (light green arrows 1' and 2').

Scale bars in (A and C), 5 µm. Error bars in (B), SE.

Table S2. UA3Es increasingly co-expressed with their AIDE counterparts during neuronal development[a]

ID	UA3E coordinates	Strand	Kendall, tau	Kendall, p value	Kendall, BH-adjusted p value	Aliases	Description	Ptbp1/2-dependent?
3200002M19Rik	chr7:109047708-109048057	+	0.91	0	0	CK051_MOUSE	RIKEN cDNA 3200002M19 gene; p	no
Fxc1	chr7:112790136-112790357	+	0.80	0	0	Tim9b,TIM9B_MOUSE,Timm9b,Fx	fractured callus expressed transcrip	no
Rbm26	chr14:105513736-105514503	-	0.73	0	0	Rbm26	RNA binding motif protein 26	no
Ppp2r1b	chr9:50687944-50689727	+	0.84	0	0	Ppp2r1b,2AAB_MOUSE	protein phosphatase 2 (formerly 2A	no
Nrp2	chr1:62842283-62843665	+	0.76	0	0	Nrp2,RP23-149A5.1-004,RP23-149	neuropilin 2	no
Sin3b	chr8:75263629-75263806	+	0.90	0	0	SIN3B_MOUSE,Sin3b		no
2610029G23Rik	chrX:102278355-102280525	+	0.79	0	0	CX026_MOUSE	WD repeat domain 43; RIKEN cDN	no
1110005A03Rik	chr11:116707077-116707447	+	0.74	0	0	uc007mml.1	predicted gene 14127; RIKEN cDN	no
Sept11	chr5:93602564-93603984	+	0.81	0	0	Sept11,SEP11_MOUSE,D5Ertd606	septin 11	yes
Rundc3a	chr11:102262703-102263068	+	0.79	0	0	O08576_MOUSE,Rundc3a,Rap2ip	RUN domain containing 3A	no
Mov10	chr3:104608326-104610020	-	0.77	0	0	Gb110,MOV10_MOUSE,Mov10	Moloney leukemia virus 10; predict	no
Nsmce2	chr15:59369082-59371219	+	0.91	0	0	NSE2_MOUSE,Mms21,Nsmce2	predicted gene 5203; non-SMC ele	no
Rexo2	chr9:48281596-48282506	-	0.79	0	0	Rexo2,ORN_MOUSE,Smfn	REX2, RNA exonuclease 2 homolo	no
Gnas	chr2:174163122-174163585	+	0.84	0	0	Gnas1,Gnas	GNAS (guanine nucleotide binding	yes
Vamp4	chr1:164524677-164525401	+	0.82	0	0	Vamp4,VAMP4_MOUSE	vesicle-associated membrane prote	no
Ewsr1	chr11:4990462-4991480	-	0.88	0	0	Ewsh,Ewsr1,RP23-338J18.1-002,R	predicted gene 6627; Ewing sarcor	no
Asnsd1	chr1:53401486-53401773	-	0.88	0	0	Asnsd1	asparagine synthetase domain con	no
Asnsd1	chr1:53401488-53401823	-	0.89	0	0	Asnsd1	asparagine synthetase domain con	no
Usp19	chr9:108403525-108404030	+	0.79	0	0	Usp19	ubiquitin specific peptidase 19	no
Socs6	chr18:89037272-89040310	-	0.75	0	0	Socs6,Cis4	suppressor of cytokine signaling 6	no
Lgtn	chr1:133068498-133068811	+	0.72	1.19E-07	1.64E-06	Lgtn,LIGA_MOUSE	ligatin	no
Tfpi	chr2:84280880-84283082	-	0.70	1.19E-07	1.64E-06	Tfpi,TFPI1_MOUSE	tissue factor pathway inhibitor	no
Rbm41	chrX:136478782-136480442	-	0.70	1.19E-07	1.64E-06	Rbm41,RBM41_MOUSE	RNA binding motif protein 41	no
Acbd6	chr1:157419328-157419418	+	0.71	1.19E-07	1.64E-06	Acbd6	acyl-Coenzyme A binding domain c	no
Cbx6	chr15:79656571-79659117	-	0.70	1.19E-07	1.64E-06	Cbx6,CBX6_MOUSE,Nptxr,Npcd	chromobox homolog 6; neuronal pe	no
Cnot4	chr6:34995176-34996358	-	0.69	2.38E-07	3.00E-06	Cnot4,Not4,CNOT4_MOUSE	CCR4-NOT transcription complex,	no
Pvt1	chr15:62080713-62082530	+	0.69	2.38E-07	3.00E-06	uc007vyl.1		yes
Cdc37l1	chr19:29087278-29089841	+	0.67	3.58E-07	4.27E-06	Cdc37l1	cell division cycle 37 homolog (S. c	no
Gigyf2	chr1:89251546-89252297	+	0.67	3.58E-07	4.27E-06	Tnrc15,PERQ2_MOUSE,Perq2,Gig	GRB10 interacting GYF protein 2	no
Lrch1	chr14:75163130-75165054	-	0.67	4.77E-07	5.28E-06	Lrch1,mKIAA1016,LRCH1_MOUSI	leucine-rich repeats and calponin h	no
Dffb	chr4:153346623-153346787	-	0.66	5.96E-07	6.42E-06	Cad,Dffb,DFFB_MOUSE	DNA fragmentation factor, beta sub	no
Wiz	chr17:32513123-32515443	-	0.65	7.15E-07	7.50E-06	wiz,Wiz	widely-interspaced zinc finger motif	no
Ncor1	chr11:62214885-62216937	-	0.65	1.07E-06	1.05E-05	RIP13,RP23-330N10.2-009,RP23-	nuclear receptor co-repressor 1	no
Ece2	chr16:20618463-20618942	+	0.64	1.55E-06	1.44E-05	Ece2	endothelin converting enzyme 2	yes
Hps1	chr19:42832571-42833797	-	0.63	2.03E-06	1.78E-05	Ep,HPS1_MOUSE,Hps1,Hps,ep	Hermansky-Pudlak syndrome 1 hor	yes

2610044O15Rik	chr17:95214149-95215193	-	0.62	2.62E-06	2.19E-05	uc008dwt.1	RIKEN cDNA 2610044O15 gene	no
Rbm5	chr9:107660775-107662106	-	0.61	3.58E-06	2.83E-05	Luca15,RBM5_MOUSE,Rbm5	RNA binding motif protein 5	no
Cntn4	chr6:106623944-106624258	+	0.63	4.53E-06	3.36E-05	CNTN4_MOUSE,Cntn4	contactin 4	no
Cdc42	chr4:136877890-136878534	-	0.60	5.01E-06	3.60E-05	CDC42_MOUSE,Cdc42	cell division cycle 42 homolog (S. c	yes
Kidins220	chr12:25741447-25744562	+	0.60	5.84E-06	4.10E-05	uc007nfc.1	kinase D-interacting substrate 220	no
Nap1l1	chr10:110932107-110932255	+	0.60	5.84E-06	4.10E-05	NP1L1_MOUSE,Nrp,Nap1l1	similar to nucleosome assembly pr	no
Zfp64	chr2:168750861-168752433	-	0.60	5.84E-06	4.10E-05	Zfp64,ZFP64_MOUSE	zinc finger protein 64	no
Gnas	chr2:174153868-174154347	+	0.60	6.91E-06	4.64E-05	Gnas,GNAS2_MOUSE,Gnas1		no
Rnf130	chr11:49918051-49918235	+	0.60	6.91E-06	4.64E-05	RP23-319B15.1-002,Rnf130,GOLI	ring finger protein 130; similar to Ri	no
Brsk2	chr7:149184825-149184919	+	0.59	8.11E-06	5.18E-05	BRSK2_MOUSE,mKIAA4256,Brsk	BR serine/threonine kinase 2	no
Asph	chr4:9548057-9549373	-	0.59	9.54E-06	5.97E-05	ASPH_MOUSE,Asph	aspartate-beta-hydroxylase	no
Myh10	chr11:68559437-68559985	+	0.60	1.00E-05	6.17E-05	mKIAA3005,MYH10_MOUSE,Myh	myosin, heavy polypeptide 10, non	no
Epha7	chr4:28873369-28874197	+	0.58	1.11E-05	6.75E-05	Ehk3,Ebk,EPHA7_MOUSE,Mdk1,E	Eph receptor A7	no
Gtf3c2	chr5:31458379-31460140	-	0.58	1.30E-05	7.74E-05	Gtf3c2,Mpv17,TF3C2_MOUSE,MF	general transcription factor IIIC, pol	no
1700037C18Rik	chr16:3905798-3906300	-	0.56	2.42E-05	1.32E-04	uc007xyx.1	RIKEN cDNA 1700037C18 gene	yes
Qk	chr17:10403045-10406223	-	0.56	2.42E-05	1.32E-04	Qka1,QKI_MOUSE,Qk1,Qk,Qki	similar to Quaking protein; quaking	no
Pdlim5	chr3:141966023-141966385	-	0.56	2.42E-05	1.32E-04	Pdlim5,Enh	PDZ and LIM domain 5	no
Obsl1	chr1:75492919-75493199	-	0.56	2.81E-05	1.51E-04	Obsl1	obscurin-like 1	no
Zfp235	chr7:24921005-24921929	+	0.55	3.27E-05	1.71E-04	Zfp235	zinc finger protein 235	no
Ivns1abp	chr1:153203367-153203928	+	0.55	3.27E-05	1.71E-04	Nd1-S,Ivns1abp	influenza virus NS1A binding protei	no
Ankhd1	chr18:36818054-36818562	+	0.55	3.80E-05	1.97E-04	Eif4ebp3,Ankhd1		no
Strbp	chr2:37438992-37439626	-	0.54	4.41E-05	2.22E-04	Strbp	spermatid perinuclear RNA binding	no
Gtl2	chr12:110796850-110799917	+	0.53	6.83E-05	3.23E-04	Gtl2		no
Lamp2	chrX:35772686-35775028	-	0.53	6.83E-05	3.23E-04	Lamp2,LAMP2	lysosomal-associated membrane p	no
E130308A19Rik	chr4:59732513-59733841	+	0.51	1.05E-04	4.62E-04	K1958_MOUSE	RIKEN cDNA E130308A19 gene	no
Gigyf2	chr1:89251556-89254359	+	0.51	1.05E-04	4.62E-04	Tnrc15,PERQ2_MOUSE,Perq2,Gi	GRB10 interacting GYF protein 2	no
Mirg	chr12:110977836-110979713	+	0.51	1.21E-04	5.19E-04	uc007pbd.1		no
Dak	chr19:10666687-10667279	-	0.51	1.29E-04	5.51E-04	Dak,DAK_MOUSE	dihydroxyacetone kinase 2 homolo	no
Cbx6	chr15:79654328-79659117	-	0.51	1.39E-04	5.78E-04	Cbx6,CBX6_MOUSE,Nptxr,Npcd	chromobox homolog 6; neuronal pe	no
Il1rap	chr16:26714787-26716652	+	0.51	1.54E-04	6.29E-04	IL1AP_MOUSE,Il1rap	interleukin 1 receptor accessory pr	no
Ncam1	chr9:49323244-49325687	-	0.50	1.59E-04	6.39E-04	O08909_MOUSE,Ncam1	neural cell adhesion molecule 1	yes
lysmd4	chr7:74369288-74369508	+	0.50	1.70E-04	6.78E-04	Lysmd4,LYSM4_MOUSE	LysM, putative peptidoglycan-bindi	no
D11Wsu47e	chr11:113555826-113555961	+	0.49	2.24E-04	8.73E-04	D11Wsu47e	DNA segment, Chr 11, Wayne Stat	no
Wdr20a	chr12:112031324-112033238	+	0.49	2.40E-04	9.24E-04	Wdr20a	WD repeat domain 20A	no
Ppm1b	chr17:85414776-85416461	+	0.48	3.34E-04	1.24E-03	ppm1b2,Ppm1b,PP2CB_MOUSE,F	similar to serine/threonine phospha	no
Wdr70	chr15:8042229-8043115	-	0.47	3.57E-04	1.30E-03	Wdr70	WD repeat domain 70	no
Ank2	chr3:126650030-126650725	-	0.47	4.07E-04	1.43E-03	Ank2	ankyrin 2, brain	no
Dnm2	chr9:21310777-21311584	+	0.45	7.68E-04	2.43E-03	Dnm2,Dyn2	dynamin 2	yes
6430550D23Rik	chr2:155826180-155827298	-	0.45	7.68E-04	2.43E-03	uc008nlv.1		no
Tshz2	chr2:169738108-169738587	+	0.45	8.11E-04	2.54E-03	Tsh2,Sdccag33l,Tshz2,Znf218,TSH	teashirt zinc finger family member 2	no

Itsn1	chr16:91870438-91871898	+	0.44	8.69E-04	2.66E-03	ITSN1_MOUSE,Ese1,Itsn1,Itsn	intersectin 1 (SH3 domain protein 1	yes
Snx12	chrX:98407607-98407685	-	0.44	8.69E-04	2.66E-03	Snx12,SNX12_MOUSE	sorting nexin 12	no
1500004A13Rik	chr3:88609211-88612194	-	0.44	8.59E-04	2.66E-03	uc008pwq.1		no
Epb4.1l1	chr2:156346974-156347947	+	0.44	9.69E-04	2.89E-03	mKIAA0338,Epb4,Epb4.1l1,E41L1	erythrocyte protein band 4.1-like 1	no
Hnrnpa2b1	chr6:51417399-51417425	-	0.44	9.82E-04	2.92E-03	hnRNP A2/B1,Hnrpa2b1,Hnrnpa2b	predicted gene 5778; similar to het	no
Zfp451	chr1:33858805-33860458	-	0.42	1.41E-03	3.83E-03	ZN451_MOUSE,Znf451,Zfp451	zinc finger protein 451	no

a) Ptbp1/2-dependent events are shaded in blue.

Table S3. GO terms enriched for Ptbp1/2-regulated genes with increasing co-expression of UA3E and AIDE during neuronal development

Term	Term ID	Fold enrichment	p-value	Benjamini-adjusted p-value	FDR	Gene count	Genes
Cell projection	GO:0042995	13.59	1.39E-04	7.04E-03	0.13	5	Cdc42, Gnas, Itsn1, Ncam1, Sept11
GTP binding	GO:0005525	21.45	3.53E-04	1.19E-02	0.31	4	Cdc42, Dnm2, Gnas, Sept11
Guanyl nucleotide binding	GO:0032561	20.92	3.80E-04	6.45E-03	0.33	4	Cdc42, Dnm2, Gnas, Sept11
Guanyl ribonucleotide binding	GO:0019001	20.92	3.80E-04	6.45E-03	0.33	4	Cdc42, Dnm2, Gnas, Sept11
Plasma membrane part	GO:0044459	5.74	6.31E-04	1.60E-02	0.61	6	Cdc42, Dnm2, Gnas, Itsn1, Ncam1, Sept11
GTPase activity	GO:0003924	44.49	1.35E-03	1.52E-02	1.18	3	Cdc42, Dnm2, Gnas

Table S4. Plasmids generated in this study

Name	Alternative name	Description	Vector	Insert or treatment
pEM305	N/A	Plasmid encoding a 3'-terminal part of mouse *Cdc42* gene including a part of I5, E6, I6, E7 and a short downstream sequence	pEM157 (Makeyev et al., 2007) cut with *Pme*I and *Spe*I	A 3'-terminal fragment of mouse *Cdc42* gene amplified with EMO148/EMO153 primers and cut with *Spe*I
pEM607	ds-E6-E7	*CMV* promoter-driven minigene containing a 3'-terminal part of Cdc42	pEGFP-N1 (Clontech) cut with *Bam*HI and *Not*I to remove the *EGFP* gene	A 3'-terminal fragment of *Cdc42* released from pEM305 with *Bam*HI and *Not*I
pEM1121	ds-E6-Red	*CMV* promoter-driven minigene containing E6 and adjacent *Cdc42*-specific sequences within a constitutive intron of *dsRed* gene	pEM157 (Makeyev et al., 2007)treated with *Spe*I, Klenow, *and Pme*I	A 3'-terminal fragment of *Cdc42* was amplified from pEM607 with EMO2693/2694 primers and cut with *Spe*I
pEM1122	ds-E6SYNpA-Red	Modified pEM1121 containing a synthetic cleavage/polyadenylation sequence (pA) in place of the endogenous *Cdc42* E6 pA	pEM157 (Makeyev et al., 2007) treated with *Spe*I, Klenow, and *Pme*I	A 3'-terminal fragment of *Cdc42* amplified from pEM607 with EMO2693/2695 and cut with *Spe*I
pEM1174	ds-E6SYNpA(iPE-mut)-Red	Mutated pEM1122 lacking the intronic Ptbp1/2 consensus element (iPE)	pEM1122	Mutagenized using EMO1868/EMO1869 primers
pEM1210	ds-E6SYNpA(ePE-mut)-Red	Mutated pEM1122 lacking the exonic Ptbp1/2 consensus element (ePE)	pEM1122	Mutagenized using EMO3081/EMO3082 primers
pEM1211	ds-E6SYNpA(iePE-mut)-Red	Mutated pEM1122 lacking both iPE and ePE	pEM1174	Mutagenized using EMO3081/EMO3082 primers
pEM1421	N/A	Modified pEM607 with the 5'ss-like sequence CAGGTGTGTGCT within the E7 ORF mutated to CAGCACACAGCT to generate E7' version of this exon	pEM607	Mutagenized using EMO4302/EMO4303 primers
pEM1118	N/A	Modified pEM607 with two *BsmB*I sites replacing a part Cdc42 E6	pEM607	Mutagenized using EMO2687/EMO2688 primers
pEM1426	ds-E7'/6-E7	Modified pEM607 where a large portion of E6 was replaced with E7'	pEM1118 cut with *Bsm*BI	E7'-containing fragment amplified from pEM1421 using EMO2719/EMO4363 primers and cut with *Bsm*BI
pEM1427	ds-E6-E6/7	Modified pEM607 where a large portion of E7 was replaced with E6	pEM607 treated with *Xcm*I, Klenow, and *Acl*I	E6-containing fragment amplified from pEM607 using EMO4364/EMO4365 primers and cut with *Acl*I
pEM1004	N/A	CMV promoter-driven minigene containing E7 and adjacent Cdc42-specific sequences within a constitutive intron of dsRed gene	pEM157 (Makeyev et al., 2007) treated with *Spe*I, Klenow, and *Pme*I	E7-containing fragment amplified from pEM607 using EMO36/EMO375 primers and cut with *Spe*I

pEM1423	ds-E7'-Red	Modified pEM1004 with the 5'ss-like sequence CAGGTGTGTGCT within the E7 ORF mutated to CAGCACACAGCT to generate E7' version of this exon	pEM1004	Mutagenized using EMO4302/EMO4303 primers
pEM1205	N/A	Plasmid containing shE6 (#4) insert	pEM791 (Khandelia et al., 2011) cut with *Bsm*BI	Annealed EMO3111/EMO3112 oligonucleotides
pEM1206	N/A	Plasmid containing shE7 (#3) insert	pEM791 (Khandelia et al., 2011) cut with *Bsm*BI	Annealed EMO3117/EMO3118 oligonucleotides
pEM1373	EGFP-shE6	Lentiviral construct for shE6 (#4) expression	pGIPZ (Open Biosystems) cut with *Bsr*GI and *Mlu*I	shE6 (#4) containing products amplified from pEM1205 using EMO4056/EMO4057 primers and cut with *Bsr*GI and *Mlu*I
pEM1375	EGFP-shE7	Lentiviral construct for shE7 (#3) expression	pGIPZ (Open Biosystems) cut with *Bsr*GI and *Mlu*I	shE7 (#3) containing products amplified from pEM1206 using EMO4056/EMO4057 primers and cut with *Bsr*GI and *Mlu*I
pEM1376	EGFP-shluc	Lentiviral construct for shLuc expression	pGIPZ (Open Biosystems) was treated with *Bsr*GI and *Mlu*I	shLuc-containing fragment amplified from pEM830#15 (Khandelia et al., 2011) with EMO4056/EMO4057 and cut with *Bsr*GI and *Mlu*I
pEM1305	YFP-Cdc42E7	Lentiviral construct containing YFP tagged CDC42E7	pEM584 (Khandelia et al., 2011) cut with *Nco*I and *Bam*HI	YFP-Cdc42E7 fragment released from modified YFP-Cdc42 plasmid (Hoppe and Swanson, 2004) with *Nco*I and B*am*HI
pEM1306	YFP-Cdc42E6	Lentiviral construct containing YFP tagged with CDC42E6	pEM584 (Khandelia et al., 2011) cut with *Nco*I and *Bam*HI	YFP-Cdc42E6 fragment released from YFP-Cdc42 plasmid (Hoppe and Swanson, 2004) with *Nco*I and B*am*HI
pEM1311	YFP vector	Lentiviral construct containing YFP only	pEM1306 cut with *Bsr*GI and *Bam*HI	Annealed EMO3961/EMO3970 oligonucleotides restoring YFP C-terminus and stop codon
pEM434	N/A	pL451 (Liu et al., 2003) modified by replacing bGHpA with SV40pA	PCR fragment amplified from pL451 (Liu et al., 2003) using EMO173/EMO172 primers and cut with *Afl*II	SV40pA fragment excised from pEGFP-N1 (Clontech) using *Xba*I, Klenow*, and Afl*II
pEM435	N/A	pL452 (Liu et al., 2003) modified by removing *PGK* promoter	PCR fragment amplified from pL452 (Liu et al., 2003) with EMO173/EMO174 and cut with *Nhe*I	PCR fragment containing EM7 bacterial promoter and downstream *Neo*^{R} gene amplified from pL452 with EMO171/EMO172 and cut with *Nhe*I
pGAP-5'+3'Cdc42	N/A	Modified *DTA*-containing gap-repair vector (Chen et al., 2012) with *Cdc42*-specific 5' and 3' homology arms	Gap-repair vector (Chen et al., 2012) cut with *Eco*RV and *Sal*I	The 5' and 3' homology arms were amplified with EMO60/EMO61 and EMO58/EMO59 primers respectively, cut with *Bgl*II and ligated. Gel-purified ligation product comprising both homology arms was then cut with *Sal*I

pEM422	N/A	Modified pGAP-5'+3'Cdc42 containing a large 3'-terminal fragment of *Cdc42*	pGAP-5'+3'Cdc42 cut with *Bgl*II	Bacterial artificial chromosome bMQ291-A10 (BACPAC/CHORI) was homologously recombined with linearized pGAP-5'+3'Cdc42 (Liu et al., 2003)
pEM431	N/A	Modified pEM422 containing *LoxP*-flanked Neo^R gene upstream of Cdc42 E6	pEM422	*LoxP-Neo-LoxP* fragment amplified from pEM435 with EMO117/EMO118 primers (introducing short Cdc42-specific homology arms specific to sequences upstream of Cdc42 E6) was homologously recombined with pEM422 (Liu et al., 2003)
pEM459	N/A	Modified pEM431 that left only a single *LoxP* site upstream of the Cdc42 homology arm	pEM431	Neo^R gene was excised from pEM431 using *Cre/LoxP* recombination (Liu et. al., 2003)
pEM461	N/A	Modified pEM459 with additional insertion of FRT-NeoR-FRT-LoxP sites downstream of Cdc42 E6	pEM459	*FRT-NeoR-FRT-LoxP* fragment amplified from pEM434 with EMO226/EMO227 primers (introducing short homology arms specific to sequences downstream of Cdc42 E6) was homologously recombined with pEM459 (Liu et al., 2003)

Table S5. Primers used in this study

Name	ID	Sequence 5' to 3'
**RT-PCR and RT-qPCR analyses**		
F1	EMO36	CGTGATGCAGAAGAAGACCA
R1	EMO2792	GTGGGACAGGAAGCAGCAG
R2	EMO37	AGCTTGGCGTCCACGTAGTA
R3	EMO2793	GCAGAAAGGGCTCTGGAGAT
R4	EMO4362	CAGGGAGCAGCTTTGACAAT
R5	EMO2801	GCAGGGCGTTTGTCATTATT
Cdc42-F1	MLO171	GGGACCCAAATTGATCTCAG
Cdc42-F2	EMO2773	TGCCAAGAACAAACAGAAGC
Cdc42_F3	EMO1463	CGACCGCTAAGTTATCCACAGA
Cdc42-F5	MLO176	CGACCGCTAAGTTATCCACA
Cdc42-R1	EMO151	GGCAGCTAGGATAGCCTCAT
Cdc42-R2	EMO152	GATGCGTTCATAGCAGCACA
Cdc42-R3	MLO175	GGCTCTTCTTCGGTTCTGGA
Cdc42-R4	EMO1464	CGCCAGCTTTTCAGCAGTCT
Cdc42-R5	MLO174	GTGGGACAGGAAGCAGCAG
Dnm2-F1	EMO4643	CCAACAACGACCCCTTCTCT
Dnm2-R1	EMO4644	CCAGCATGGAGGGCAAGTA
Dnm2-R2	EMO4645	CTCCCACGAAGCTCAGAAGA
Fxc1-F1	EMO4619	CCTGCAGAACAGACCAGAGA
Fxc1-R1	EMO3359	CTTCCCCTAGGTTCACAGCA
Fxc1-R2	EMO4551	GAAGCTCCTGGTCAGCAAGT
Gapdh-F1	MLO194	TGGTCACCAGGGCTGCCATT
Gapdh-F2	MLO87	AAATGGGGTGAGGCCGGTGC
Gapdh-R1	MLO195	GAGCCCTTCCACAATGCCAAA
Gapdh-R2	MLO88	ATCGGCAGAAGGGGCGGAGA
Gfap-F1	MLO182	GCCACCAGTAACATGCAAGA
Gfap-F2	MLO184	CAAGCCAAGCACGAAGCTAA
Gfap-R1	MLO183	CGATGTCCAGGGCTAGCTTA
Gfap-R2	MLO185	CATTTGCCGCTCTAGGGACT
Gnas-F1	EMO3354	GAGTCTGGCAAAAGCACCAT
Gnas-R1	EMO3355	GTTTCCTAAGACCGGGCAAT
Gnas-R2	EMO3356	GCCTTGGCATGCTCATAGAA
Itsn-F1	EMO4529	CCAGATCATCAACGTCCTCA
Itsn-R1	EMO4607	CAGTAGGTGATCATGCTGCAA
Itsn-R2	EMO4609	CTTGCCGCTTCCTCTCAGT
Ncam1-F1	EMO3342	CTCTGAGTGGAAACCGGAAA
Ncam1-R1	EMO3343	CGCAGAGAAAAGCAATGAGA
Ncam1-R2	EMO3344	CAGGTTAACAGCGATGCACA
NeuN-F1	MLO133	GGATGGATTTTATGGTGCTGA

NeuN-F2	MLO134	CAGATATGCTCAGCCAGCAG
NeuN-R1	MLO135	CCGATGCTGTAGGTTGCTGT
Nsmce2-F1	EMO4561	GGATAAGAACTCTGATGCCGACT
Nsmce2-R1	EMO4562	GGTTTTGCTTCAGAATTACTGGTT
Nsmce2-R2	EMO4563	CACTGGCTTCTTCATTTCCA
Pbdc1-F1	EMO4555	GGGACTTTACTGCGACTGGA
Pbdc1-R1	EMO4556	CAGCTGAGTCACGGGTTTCT
Pbdc1-R2	EMO4557	GAAGACTGGAGAGGGCAGAG
Ptbp1-up5	EMO863	AGTGCGCATTACACTGTCCA
Ptbp1-down5	EMO864	CTTGAGGTCGTCCTCTGACA
Ptbp2-u23	EMO91	GGAACTGGCAACAGAGGAAG
Ptbp2-d23	EMO111	TGTGGTGCCACTAAGAGGTG
Sept11-F1	EMO4536	GAGGAGGTGAGCAACTTCCA
Sept11-R1	EMO4537	CAAATCGACTTTCGAGAAACA
Sept11-R2	EMO4538	GCGATGGTGGAGATGAGGTA

Cloning and site-directed mutagenesis

Cdc42-up3	EMO148	CCTCCCACCCTCTGGTTTCTTTT
Cdc42-down3	EMO153	CGTTAATACTAGTAAGCTGGGGCAATCAGTCTA
Southern_probe2	EMO375	GCAGAACTGCTTCCCATGTT
Cdc42_mut5-F1	EMO1868	CCTCTAACCTGGCTGCTATTTTTTTTCCTCCCCTCTGTCTTGTAGAGAGG
Cdc42_mut5-R1	EMO1869	CCTCTCTACAAGACAGAGGGGAGGAAAAAAAATAGCAGCCAGGTTAGAGG
Cdc42_insBsmBI-F1	EMO2687	GCTGCTATTCTCTCTCCTCCCCGAGACGTACAGATGCGTCTCCCGTTTTCTCCTTCCCCTCTTTGC
Cdc42_insBsmBI-R1	EMO2688	GCAAAGAGGGGAAGGAGAAAACGGGAGACGCATCTGTACGTCTCGGGGAGGAGAGAGAATAGCAGC
Cdc42_I5-F1	EMO2693	TTCCCCTTGAGATTTTAAACCA
Cdc42_I6_SpeI-R1	EMO2694	GGACTAGTGCTTCACTCGGTTGTCTTGT
pGL_polyA-R1	EMO2695	GGACTAGTACTATCGATTCACACAAAAAACCAACACACAGATGTAATGAAAATAAAGATATTTTATTACACTCTACTAGCAAGCCA
Cdc42_insE6/7-F1	EMO2719	TTCCCCTTGAGATTTTAAACCA
Cdc42_mut16-F1	EMO3081	GGAAGTGCTGTATATTCTAAACCGTTTTTTTTTTTTTTTTTTTGCTGCTGCTTCCTGTCCCACTA
Cdc42_mut16-R1	EMO3082	TAGTGGGACAGGAAGCAGCAGCAAAAAAAAAAAAAAAAAAACGGTTTAGAATATACAGCACTTCC
YFP_stop-F1	EMO3961	GTACAAGTAAAGCGGCCGCTGAACTAGTTAGTTCGAATGAG
YFP_stop-R1	EMO3970	GATCCTCATTCGAACTAACTAGTTCAGCGGCCGCTTTACTT
mCdc42_I6/E7-R3	EMO4363	AAAGGGCCGTCTCCAACGGTTCATAGCAGCTGTGTGCTG
Cdc42_I5-F1	EMO4364	AAAGGGCCGTCTCCAACGGTTCATAGCAGCTGTGTGCTG
Cdc42_E6_AclI-R1	EMO4365	GAAGAACGTTCAGCAAAGAGGGGAAGGAGAA
Cdc42_mut19-F1	EMO4302	CTCCAGAACCGAAGAAGAGCCGCAGCACACAGCTGCTATGAACGCATCTCCAGA
Cdc42_mut19-R1	EMO4303	TCTGGAGATGCGTTCATAGCAGCTGTGTGCTGCGGCTCTTCTTCGGTTCTGGAG
shCdc42_E6-F4	EMO3111	TGCTGACAAACAGCCAAAGCAAGAGCGTTTTGGCCACTGACTGACGCTCTTGTTGGCTGTTTGT
shCdc42_E6-R4	EMO3112	CCTGACAAACAGCCAACAAGAGCGTCAGTCAGTGGCCAAAACGCTCTTGCTTTGGCTGTTTGTC
shCdc42_E7-F3	EMO3117	TGCTGAGTTCCATCACAGACTGGACCGTTTTGGCCACTGACTGACGGTCCAGTGTGATGGAACT
shCdc42_E7-R3	EMO3118	CCTGAGTTCCATCACACTGGACCGTCAGTCAGTGGCCAAAACGGTCCAGTCTGTGATGGAACTC
pEM791_BsrG1-F1	EMO4056	GTCGATGTACAGAGCTCTGGAGGCTTGCT
pEM791_Mlu1-R1	EMO4057	AGCGCACGCGTCGGCCATTTGTTCCATGT

DNA template for in vitro transcription

T7_Cdc42_I5-F1	EMO2425	CGAAATTAATACGACTCACTATAGGGAGCTAGTCTCTCTAATCCTCT
Cdc42_E6-R1	EMO2426	CTTTCTACAGTAGTGGGACAGGAAGCA
**Cdc42 E6 knockout mouse**		
Cdc42_gap3'-F1	EMO58	GGTAGATCTCCTCCGTCTTTTACTTTTCAGGT
Cdc42_gap3'-R1	EMO59	GTGCGTCGACTGAGCCCCTTTGCTTAGTTC
Cdc42_gap5'-F1	EMO60	GTAAAGCTTATGGATGGTGGATGCCTTC
Cdc42_gap5'-R1	EMO61	GGAAGATCTAGATGAACATGGCGGAGCTA
Cdc42_LoxP-F1	EMO117	AAGGGGTGTCGTCATCATCAATAGTAATGTTGGGGGGAATCTCACTTTTCTAACATATCTATCTTGTGTTTATAGAATTCCTGCAGCCCAATTCCGA
Cdc42_LoxP-R1	EMO118	GTATGTTAATTTCTAAAAGAATCACATACTGAAATCTAATATTACATTTTGGTTTAAAATCTCAAGGGGAAAAAATAACTAGTGGATCCCCTCGAGGGA
Neo-F1	EMO171	CGACCTGCTAGCTGTTGACAATTAATCATC
Neo-R1	EMO172	GAGAATTGATCCCCTCAGAAGAACTCGT
pL452-F1	EMO173	GCTCCTTAAGAGCTTGCGGAACCCTT
pL452-R1	EMO174	GAATTTCGACGACCTGCAGCCAA
Cdc42_FRT_LoxP_FRT-F1	EMO226	CTTTGTCTAATTAGTGGGATAAAGGGAGTTCAAGGTGATTATATTTACAGCTGCCATACCCTCGCTGTCCACTCCCTCGAGGTCGACGGTAT
Cdc42_FRT_LoxP_FRT-R1	EMO227	GTTCTGCATTCAAATGGAGCGGCAGCATGTGAAATAGAGAATACATCCGGGCAGCATCCATGTTGTATGGAAGGCCGCTCTAGAACTAGTGGA
Cdc42_southern_probe-F3	EMO1723	CATGGCATGCCCATACATAC
Cdc42_southern_probe-R3	EMO1724	GACATCAACATCTAACACATTTTGG

Table S6. Primary antibodies used in this study

Antibody	Host	Application[a]	Dilution	Source
mAb-Ptbp1 (Clone1)	mouse	WB	1:1000	Life Technologies
mAb-GFP	mouse	WB	1:1000	Life Technologies
pAb-p44/42 MAPK (Erk1/2)	rabbit	WB	1:1000	Cell Signaling
mAb-Cdc42(Clone 44/CDC42)	mouse	WB	1:50	BD Biosciences
pAb-Cdc42(C-terminus of the neuron-specific isoform Cdc42E6)	rabbit	WB	1:50	LifeSpan BioSciences
mAb-β tubulin	mouse	WB	1:1000	Life Technologies
mAb-Nestin (Clone Rat 401)	mouse	IF	1:200	StemCell Technologies
mAb-SMI312	mouse	IF	1:500	Covance
mAb-Tau-1	mouse	IF	1:500	Millipore
pAb-Map2	chicken	IF	1:1000	Covance
mAb-AnkG (Clone N106/36)	mouse	IF	1:200	NeuroMab
pAb-Homer1	rabbit	IF	1:200	Synaptic Systems
mAb-PSD95 (Clone 7E3-1B8)	mouse	IF	1:100	Thermo Scientific

a) WB, Western Blotting; IF, Immunofluorescence

Supplemental Experimental Procedures

Bioinformatics

RNA-seq reads were aligned with the mm9 genome and splice junctions using ExpressionPlot (Friedman and Maniatis, 2011) and normalized numbers of reads per kilobase were calculated for UA3Es ($rpkm_{UA3E}$) and AIDEs ($rpkm_{AIDE}$). In cases when a UA3E partially overlapped with a known alternative exon, $rpkm_{UA3E}$ was determined from the reads aligning with a non-overlapping 3'-terminal part of UA3E. ExpressionPlot was also utilized to quantify developmental changes in marker gene expression levels. ψ_{UA3E} values were computed as $100\times rpkm_{UA3E}/(rpkm_{UA3E}+rpkm_{AIDE})$. To identify significantly regulated UA3Es we used Kruskal-Wallis rank sum test (Hollander and Wolfe, 1973). 426 events with BH-adjusted p-values <0.005 and differences between the maximal and minimal ψ_{UA3E} values ≥10% were shortlisted for further analyses. UA3Es significantly regulated in CAD cells in response to siPtbp1 and siPtbp1/2 (≥1.5-fold change and BH-adjusted p value <0.05 for both treatments) were extracted from our published RNA-seq dataset (Yap et al., 2012) using the 4-way comparison routine of ExpressionPlot (Friedman and Maniatis, 2011).

Enrichment of RBP motifs was analyzed in 250-nt sequence windows centered on the 3'ss position. For this purpose, we computed GC-compensated average motif affinity (AMA) p-values for 95 high-quality *Mus musculus* position weight matrices from the CisBP-RNA database (Ray et al., 2013) using corresponding program of the MEME suite (Buske et al., 2010). Since AMA p-values are inversely correlated with motif occurrence in a sequence, motifs with significantly smaller AMA p-values for the 426 regulated UA3Es compared to the 769 non-regulated ones (BH-adjusted one-sided Kolmogorov-Smirnov test p<0.05) were considered enriched (see Table S1).

Data bimodality in Fig. 1B was tested using unrestricted likelihood ratio test [R package bimodalitytest; (Holzmann and Vollmer, 2008); http://www.R-project.org/]. Kernel density estimates in Fig. 1B were generated using the density function in R with the bandwidth of 0.2. Gene Ontology terms were analyzed using DAVID [http://david.abcc.ncifcrf.gov/; (Huang et al., 2009)].

We defined isoform co-expression index (ν) similarly to the N_1 statistic used in ecology to estimate effective number of species (Hill, 1973)

$$\nu = \exp(H)$$

where

$$H = -\sum_{i=1}^{n} p_i \ln(p_i)$$

is Shannon's entropy for n different species occurring in the sample with proportional abundances $p_1, p_2, \ldots, p_n$. Note that if one or several $p_i = 0$, corresponding $0\ln(0)$ elements are assigned 0 value, since $\lim_{p\to 0+} p\ln(p) = 0$.

For two alternative isoforms UA3E and AIDE, $n = 2$ and the proportional abundances are $p_{\mathrm{UA3E}} = \psi_{\mathrm{UA3E}}/100$ and $p_{\mathrm{AIDE}} = 1 - p_{\mathrm{UA3E}}$, respectively. In this case,

$$\nu = \exp(-p_{\mathrm{UA3E}} \ln(p_{\mathrm{UA3E}}) - (1 - p_{\mathrm{UA3E}}) \ln(1 - p_{\mathrm{UA3E}}))$$

Since $p_{\mathrm{UA3E}} \in [0, 1]$, $\nu \in [1, 2]$ and is minimal $\nu_{min} = 1$ at $p_{\mathrm{UA3E}} = 0$ or $p_{\mathrm{UA3E}} = 1$ and maximal $\nu_{max} = 2$ at $p_{\mathrm{UA3E}} = 0.5$.

Generally, for any number of splicing alternatives ($n \geq 1$), $\nu \in [1, n]$ reaching $\nu_{min} = 1$ when only one (i^{th}) alternative is realized (i.e. $p_i = 1$) and $\nu_{max} = n$ when all n alternatives occur with equal proportional abundances (i.e, $p_1 = p_2 = \cdots = p_n = \frac{1}{n}$). Therefore, ν provides an intuitive estimate of isoform co-expression in an AS mixture.

DNA constructs

Plasmids pEGFP-N1 and pGIPZ were obtained from Clontech/TaKaRa and Open Biosystems/GE Healthcare, respectively, and bacterial artificial chromosome bMQ291A10 encoding mouse *Cdc42* gene was from BACPAC Resource Center (CHORI, Oakland, CA). Plasmids YFP-Cdc42, recombineering gap-repair vector containing a *DTA* marker, pL451, pL452, pEM157, pEM584, pEM791 (pRD-RIPE), and pEM830#15 (pRD-RIPE-shLuc) were described previously (Chen et al., 2012; Hoppe and Swanson, 2004; Khandelia et al., 2011; Liu et al., 2003; Makeyev et al., 2007). New constructs (Table S4) were prepared using routine molecular approaches (Sambrook et al., 2001) and restriction and modification enzymes from New England Biolabs. PCR amplification and site-directed mutagenesis were carried out using HiFi DNA polymerase (KAPA Biosystems) and primers listed in Table S5. miR-155 based shRNAs were designed as described (Khandelia et al., 2011). shRNA-encoding oligonucleotide pairs were annealed at 10 μM concentration in 50 mM NaCl, 10 mM Tris-HCl, pH 7.5 and 1 mM EDTA. The mixtures

were incubated at 95°C for 5 min, allowed to gradually cool down to room temperature and ligated with linearized vector DNA. All constructs were confirmed by sequencing and their detailed maps are available on request.

Cell lines and lentiviral stocks

CAD neuroblastoma cell line (Qi et al., 1997) was propagated in high-glucose Dulbecco's Modified Eagle Medium (DMEM; HyClone/GE Healthcare) additionally containing 11% FetalClone III serum (HyClone/GE Healthcare), 100 units/ml penicillin, 100 μg/ml streptomycin (GIBCO/Life Technologies) and 1 mM sodium pyruvate (GIBCO/Life Technologies) at 37°C, 5% CO_2. HEK293T cells were maintained in DMEM supplemented with 10% fetal bovine serum (FBS; HyClone/GE Healthcare), 100 units/ml penicillin, 100 μg/ml streptomycin and 1 mM sodium pyruvate.

For RNA interference and minigene expression experiments, 1×10^5 CAD cells were plated per well of a 12-well plate in 1 ml of CAD medium without antibiotics and allowed to attach overnight. Next morning, cells were transfected with 50 nM of corresponding ON-TARGETplus siRNAs (Dharmacon/GE Healthcare) using Lipofectamine 2000 (Life Technologies) as recommended. Cells were harvested 72 hours post transfection or alternatively re-transfected at the 48-hour time point with 0.8 μg of minigene DNA and incubated for another 24 hours prior to further analyses.

To produce lentiviral vector stocks, HEK293T cells were co-transfected with an appropriate vector construct (Table S4) and Lenti-X HT packaging mixture containing pVSV-G, pTre-GAG-PRO, pLR2P-vpr-RT-IN, pTet-Off and pTre-Tat-IRES-Rev plasmids (Clontech/TaKaRa) using TransIT®-293 transfection reagent (Mirus Bio LLC). Lentiviral particle-containing medium was typically harvested 48 hours post-transfection, and cleared from cell debris by passing through 0.45 μm low protein binding filters (PALL Life Sciences). Particles were concentrated using Lenti-X Concentrator solution (Clontech/TaKaRa) as recommended and resuspended in neuronal maintenance medium [MEM with L-glutamine, 0.6% glucose and 1× Neurocult SM1 neuronal supplement (STEMCELL Technologies)].

Primary cells

To prepare NSC cultures, E14 mouse embryonic cortices were dissected in Hank's Balanced Salt Solution (1×HBSS; Life Technologies) and dissociated by mechanical trituration. The NSCs were then cultured as neurospheres in reconstituted NeuroCult® Proliferation Kit medium supplemented with 20 ng/ml recombinant human EGF (STEMCELL Technologies). For passaging, neurospheres were dissociated with NeuroCult® Chemical Dissociation Kit (STEMCELL Technologies) as recommended. Adherent NSC cultures were established by plating dissociated NSCs onto polyornithine/fibronectin-coated surfaces.

Primary cortical neurons were isolated from E15.5 mouse embryos and hippocampal neurons were from E17.5-E18.5 mouse embryos and cultured as described (Kaech and Banker, 2006). Briefly, cortices or hippocampi were dissociated with 2.5% trypsin (GIBCO/Life Technologies) and plated onto 18 mm coverslips pretreated with poly-L-lysine (Sigma) in Minimum Essential Media (MEM) with L-glutamine (Life Technologies), 0.6% glucose (Sigma) and 10% horse serum (GIBCO/Life Technologies) at a typical density of 2.5×10^4 neurons per coverslip. Neurons attached to coverslips were then transferred to wells containing a monolayer of newborn rat astrocytes in neuronal maintenance medium [MEM with L-glutamine, 0.6% glucose and 1× Neurocult SM1 neuronal supplement (STEMCELL Technologies)] and cultured for up to 1 month replacing half of the medium every 3-4 days. Short-term neuronal cultures were occasionally maintained without glial feeders on surfaces coated with 30 μg/ml poly-D-lysine (Sigma) and 2 μg/ml laminin (Sigma). For transduction experiments, neurons were incubated for 24 hours with lentiviral particles suspended in neuronal maintenance medium.

To prepare type-I astroglia for single-cell gene expression analyses, cortices of newborn mouse pups were dissociated with 2.5% trypsin and 1 mg/ml DNase as described (Kaech and Banker, 2006) and plated in MEM with L-glutamine (Life Technologies) additionally supplemented with 0.6% glucose, 10% FBS (Hyclone), 100 units/ml penicillin and 100 μg/ml streptomycin (Life Technologies) at 2×10^6 cells per well of a 6-well plate. Medium was changed once after 3 days and the cultures were maintained for a total of 7 days to allow astrocytes to expand to ~90% confluence.

Knockout mice

Cdc42 E6 targeting construct (pEM461) was prepared as outlined in (Liu et al., 2003) and Table S4, linearized with *Pvu*I and electroporated into W4 (129S6/SVEvTac) mouse ESCs (Taconic) as described (Nagy, 2003). ESCs were then plated onto mitomycin C-treated PMEF-NL feeders (EmbryoMax/Merck Millipore) in ESC medium [Knockout™ DMEM, 2 mM L-glutamine, 1×MEM nonessential amino acids (Life Technologies), 0.1 mM β-mercaptoethanol (Chemicon/Merck Millipore), 1 mM sodium pyruvate, 100 units/ml penicillin, 100 μg/ml

streptomycin, 15% ES cell-qualified FBS (Life Technologies) and 1000 units/ml ESGRO® Leukemia Inhibitory Factor (Chemicon/Merck Millipore)]. Recombinant ESC clones resistant to 200 μg/ml G418 were validated by Southern blotting and used to generate chimeric mice as described (Nagy, 2003).

Heterozygous offspring of male chimeras and C57BL/6J females was crossed with C57BL/6J *Tg(Prm-cre)58Og* transgenes containing *Cre* recombinase gene under the protamine 1 promoter to produce animals heterozygous (HZ) for the *Cdc42*$^{tm1.2Mkv}$ allele with the *Cdc42* exon 6 replaced by a *LoxP* sequence. These were further crossed for 5 generations with wild-type C57BL/6J mice and interbred to obtain *Cdc42*$^{tm1.2Mkv/tm1.2Mkv}$ homozygotes (KO).

Mice were maintained under specific pathogen-free conditions, 12 hours light - 12 hours dark cycle and standard chow available ad libitum. All animal procedures were approved by the Institutional Animal Care and Use Committee and the Home Office.

Southern blotting

ESCs were dissociated by 0.25% trypsin/EDTA (Life Technologies) and plated in the ESC medium for 30 min at 37°C to allow selective attachment of feeder cells. Feeder-depleted ESCs were then spun down at 500 rpm for 5 min, resuspended in gDNA lysis buffer and incubated overnight at 55°C with gentle agitation. The lysates were extracted with Tris-saturated phenol (Life Technologies), phenol-chloroform (1:1) and chloroform. Genomic DNA was then precipitated with 2 volumes of ethanol, washed with 70% ethanol, air-dried and rehydrated in 10 mM Tris-HCl, pH 8.0. In some experiments, genomic DNA was prepared by incubating mouse liver samples in gDNA lysis buffer followed by the purification steps described above.

Fifteen μg of genomic DNA was incubated overnight at 37°C in 200 μl reaction mixtures containing 70 units of an appropriate restriction enzyme, extracted once with phenol-chloroform (1:1), precipitated with ethanol and rehydrated in 20 μl of 10 mM Tris-HCl, pH 8.0. The samples were then separated in 0.8% agarose gels containing 1×TAE buffer at 5 V/cm and transferred to a Hybond N+ membrane (GE Healthcare) as described (Sambrook et al., 2001). Cdc42-specific fragments were detected using a denatured double-stranded DNA probe amplified with KAPA HiFi DNA polymerase and EMO1723/EMO1724 primers (Table S5), labeled using Megaprime DNA labeling system (GE Healthcare) and [α-^{32}P]-dCTP (Perkin Elmer) and passed through G-50 spin columns (Geneaid Biotech). Hybridization was carried out in ExpressHyb hybridization buffer (Clontech/TaKaRa) as recommended and the radioactive bands were visualized using a Typhoon Trio Imager (GE Healthcare).

PCR genotyping

For routine mouse genotyping, ~0.5 cm tail biopsies were incubated in 200 μl gDNA lysis buffer (100 mM Tris-HCl, pH 8.0, 200 mM NaCl, 5 mM EDTA, 0.2% SDS, 0.2 mg/ml Proteinase K) at 55ºC overnight with continuous agitation. Proteinase K was inactivated at 95°C for 5 min and genomic DNA was precipitated from the lysates with 200 μl of isopropanol, washed once with 70% ethanol and rehydrated in 70 μl of TE buffer. The samples were then analyzed by multiplex PCR using KAPA Taq polymerase (KAPA Biosystems) and EMO152/EMO183/EMO184 primers (Table S5).

Routine RT-PCR and RT-qPCR analyses

Total RNA was purified from cells and tissues using Trizol (Life Technologies) as recommended with an additional acid phenol-chloroform (1:1) extraction step. RNA samples were treated with 50-100 units/ml of RQ1 DNase (Promega) at 37°C for 30 min to remove traces of genomic DNA. Reverse transcription (RT) was carried out using SuperScript III (Invitrogen) and random decamer (N10) primers at 50°C for 90 min. cDNA samples were analyzed by PCR using KAPA Taq DNA polymerase or quantitative PCR (qPCR) using KAPA SYBR Fast qPCR Master Mix (KAPA Biosystems) and primers listed in Table S5. RT-PCR products were resolved by electrophoresis in 2% agarose gels. RT-qPCR reactions were carried out in triplicate using a StepOnePlus Real-Time PCR System (Applied Biosystems). RT-qPCR signals were typically normalized to Gapdh mRNA expression levels (primers Gapdh-F2/Gapdh-R2; Table S5).

Single-cell gene expression analyses

Hippocampi dissected from newborn C57BL/6 mouse pups were dissociated with 2.5% trypsin, triturated in FACS buffer (1×HBSS, 10 mM HEPES, pH 7.3 and 2% FBS) to obtain a single-cell suspension and stained with 1 μg/ml of Hoechst 33342 (Life Technologies) for 15 min at 37°C. Adherent astrocyte cultures prepared as described above were incubated with 1 μg/ml of Hoechst 33342 for 15 min at 37°C, detached with 0.05% trypsin-EDTA (Life Technologies), centrifuged at 200×g for 5 min and resuspended in the FACS buffer.

Both hippocampal and astrocyte cell suspensions were passed through 70 µm strainers (BD Biosciences) and sorted using a BD FACSAria II into 96-well PCR plates (Bio-Rad) containing 4 µl Single-Cell Lysis Solution combined with DNase I, as recommended (Single Cell Lysis Kit; Life Technologies). Cells were separated from debris by selecting the corresponding population in the forward scatter (FSC-A) vs. side scatter (SSC-A) plot. Two additional gates were applied to ensure that a single living cell is deposited per well: (1) selecting singlets and excluding cell clusters using a FSC-A vs. FSC-H plot and (2) further choosing Hoechst-positive living cells with fluorescence levels corresponding to a single complement of genomic DNA (a majority of the FSC-A/FSC-H-gated singlets).

Cell lysates were reverse-transcribed with SuperScript VILO enzyme mixture (Life Technologies), as recommended and the resultant cDNAs were amplified by two rounds of PCR. In the first PCR, cDNAs from single cells were amplified in 30 µl reactions containing 1×PCR buffer, 0.3 mM of each dNTP, 2 mM $MgCl_2$, 2 units of Platinum Taq (Life Technologies) and a mixture of gene-specific primers: Gapdh-F1, Gapdh-R1, NeuN-F1, NeuN-R1, Gfap-F1, Gfap-R1, Cdc42-F5, Cdc42-R3 and Cdc42-R5 (0.15 µM each; Table S5). The following PCR program was used: initial denaturation at 94°C for 3 min followed by 30 cycles of denaturation at 94°C for 30 s, annealing at 58°C for 45 s and extension at 72°C for 50 s. The second Platinum Taq PCR was typically carried out in 20 µl reactions containing 0.5 µl of the first-round PCR products and corresponding gene-specific primers blended to amplify a housekeeping control (Gapdh-F2 and Gapdh-R2), a neuronal or an astrocyte-specific marker (NeuN-F2, NeuN-R1, Gfap-F2, and Gfap-R2), or the two splice isoforms of Cdc42 mRNA (Cdc42-F1, Cdc42-R3, and Cdc42-R5) (Table S5). The program used for the second round of PCR consisted of initial denaturation at 94°C for 3 min followed by 33 cycles of denaturation at 94°C for 30 s, annealing at 60°C for 45 s and extension at 72°C for 20 s RT-PCR products were analyzed by electrophoresis in 2% agarose gels.

Cdc42 isoform expression was additionally examined in primary hippocampal neurons and astrocytes using Quasar 570- (Cdc24E6) Quasar 670-labeled (Cdc42E7) RNA FISH Stellaris probe sets (Biosearch Technologies) and the protocol recommended by the manufacturer.

Biotinylated RNA/protein pull-down assays

Biotinylated RNAs were prepared by in vitro transcription of DNA fragments amplified from pEM1122, pEM1174, pEM1210 and pEM1211 (Table S5) with KAPA Taq and EMO2425/EMO2426 primers (Table S5). Two µg of DNA template was incubated with 20 units of T7 RNA polymerase (Promega), 40 units of rRNasin (Promega) and 1× biotin RNA labeling mixture (Roche) in 20 µl for 2 h at 37°C. The reactions were stopped by adding 2 units of RQ1 DNAse (Promega) and incubated for another 15 min at 37°C. Biotinylated RNAs were extracted with acid phenol-chloroform (1:1), precipitated with ethanol and rehydrated with diethylpyrocarbonate (DEPC)-treated water (Life Technologies).

One µg of biotinylated RNA was incubated with 150 µg of HeLa S3 nuclear extract in the presence of 0.8 mM ATP, 16 mM creatine phosphate, 1.6 mM $MgCl_2$, 160 ng/µl yeast tRNA (Life Technologies), 2 µg/µl heparin (Sigma) and 1.6 units/µl rRNasin for 20 min at 30°C. The reactions were then incubated with 10 µl of Streptavidin Sepharose (Sigma) for 1 h at 4°C with continuous rotation, followed by three washes with 20 mM HEPES-KOH, pH 7.9, 100 mM KCl, 20% glycerol (Promega), 0.2 mM ethylenediaminetetraacetic acid (EDTA) and 0.5 mM dithiothreitol (DTT; Life Technologies). The proteins were then eluted by boiling the beads with 20 µl of 1× SDS-PAGE loading buffer (62.5 mM Tris-HCl, pH 6.8, 2% SDS, 5% β-mercapthoethanol, 10% glycerol and 0.01% bromophenol blue) for 5 min. The eluted proteins were then analyzed by immunoblotting.

Electrophoretic mobility shift assay (EMSA)

RNA probes corresponding to wild-type intronic (iPE; 5'-UGCUAUUCUCUCUCUCCCCC-3') and exonic (ePE; 5'-CCGUUUUCUCCUUCCCCUCUUUGCUGC-3') pyrimidine-rich clusters, as well as a "scrambled" control with nucleotide composition identical to iPE but lacking strong Ptbp1 motifs (5'-UGCUUUCUACCUUCCCUCCC-3') were from Dharmacon/GE Healthcare. These were labeled in 20 µl reactions containing 2 µM RNA probe, 37.5 µCi of [γ-^{32}P] ATP (Perkin Elmer, 6000 Ci/mmol, 150 mCi/ml), 5 units of T4 polynucleotide kinase (T4 PNK; New England Biolabs) and 1× T4 PNK buffer at 37°C for 40 min. Labeled RNA probes were passed through G-25 spin columns (Geneaid) equilibrated with DEPC-treated water. EMSA was carried out using a protocol modified from (Amir-Ahmady et al., 2005). Briefly, 8 µl of 20 mM HEPES-KOH, pH 7.9, 100 mM KCl, 1 mM DTT, 20% glycerol, 0.02% NP-40 (Sigma), 100 ng/µl yeast tRNA and 6 mM $MgCl_2$ was incubated with 1 µl of purified recombinant Ptbp1 (final concentration 0.1 to 2 µM) for 8 min at 30°C with agitation. The mixture was supplemented with 1 µl of RNA probe (final specific radioactivity 50,000 cpm/µl) and incubated for another 20 min at 30°C. The reactions were chilled on ice for 5 min followed by adding 1.7-17 ng/µl of heparin. RNA-protein complexes were analyzed in 8% native polyacrylamide gel and visualized using a Typhoon Trio Imager.

Immunoblotting

Cells were washed with ice-cold phosphate-buffered saline (1×PBS) and proteins were extracted with 20 mM Tris-HCl, pH 7.5, 150 mM NaCl, 5 mM EDTA, 10% glycerol, 1% NP-40, 1 mM phenylmethanesulfonylfluoride (PMSF) and the recommended amount of cOmplete EDTA-free protease inhibitor cocktail (Roche). Protein concentration was determined using a BCA Protein Assay Kit (Pierce/Thermo Scientific). Samples were separated by 4-20% gradient SDS-PAGE (Thermo Scientific or Bio-Rad), electrotransferred to nitrocellulose or polyvinylidene difluoride (PVDF) membranes and analyzed using an appropriate primary antibody (Table S6), mouse- or rabbit-specific secondary antibody conjugated with horseradish peroxidase (GE Healthcare) and enhanced chemiluminescence reagents from Thermo Scientific or Merck Millipore.

Immunofluorescence and image analysis

Neurons were fixed on poly-L-lysine coated coverslips for 15 min with 4% paraformaldehyde (Ted Pella) and washed with 1×PBS. Cells were then permeabilized with 0.1% Triton X-100 in 1×PBS for 5 min, incubated with a blocking buffer containing 10% horse serum, 10% goat serum (HyClone/GE Healthcare) and 1% bovine serum albumin (Sigma) for 1 h at room temperature and then for another 16-18 hours at 4°C with primary antibodies (Table S6) diluted in the blocking buffer. The coverslips were washed thrice with 1×PBS and incubated for 1 hour at room temperature with appropriate Alexa-conjugated secondary antibodies (Molecular Probes/Life Technologies). Images were taken using a Zeiss LSM710 or a Nikon A1 inverted Eclipse Ni-E confocal microscopes or a Nikon Eclipse Ti epifluorescence microscope.

To analyze axons, DIV3 hippocampal neurons were stained with SMI312- or Tau1- or specific antibodies and imaged using a 20× EC Plan-Neofluar objective. At DIV14 and DIV18, axons were defined as neurites containing an AnkG-positive axon initial segment (AIS). For dendritic spine analyses, DIV21 hippocampal neurons were stained for Homer or PSD95 markers and imaged at 0.2 µm Z intervals using a 100× EC Plan-Neofluar, 1.3 NA oil immersion objective. Z-stacks were then thresholded using ImageJ (http://imagej.nih.gov/ij/) and post-synaptic puncta were quantified in randomly selected dendritic fragments thinner than 1.5 µm (2-5 fragments per neuron) using the "analyze particles" application of ImageJ (Rasband, 1997).

Distributions of neuronal categories containing different number of axons were compared using Pearson's chi-squared test for count data (R; http://www.R-project.org/). The occurrence of neuronal categories containing specific number of axons was also compared between different conditions using Student's two-tailed t-test assuming unequal variances (R; http://www.R-project.org/) and neurons prepared typically from 3 independent litters. Dendritic spine densities were compared using Student's two-tailed t-test assuming unequal variances with individual density measurements done for secondary dendritic segments >10 µm in length and using neurons derived from at least 3 independent litters.

Imaging neurons in sparsely labeled hippocampal slices

Organotypic slice cultures were prepared from isolated hippocampi of P5-P6 wild-type and Cdc42E6 null mutant mice as described (Gogolla et al., 2006; Yuan et al., 2015). Slices were transfected at 2-3 DIV using a biolistic gene gun (Bio-Rad, Hercules, CA). Briefly, gold particles (1.0 µm in diameter) were coated with the pCAG-MCS2-EGFP plasmid (Yuan et al., 2015) and immobilized onto the inner wall of Tefzel tubing (Bio-Rad). The tubing was cut into individual cartridges each containing approximately 0.1 mg of coated gold particles. Particles were then biolistically delivered into the slices using 150-180 psi of helium gas and the slices were maintained for another 2 days prior to confocal imaging of EGFP-labeled neurons. Some slices were stained with AnkG-specific antibodies before imaging to visualize AISs. Spine density was calculated for randomly selected >10 µm-long segments of secondary dendritic branches. Primary and major secondary branches initiated at ≤10 µm from the soma were classified as spine-containing or devoid of spines based on visual inspection of confocal image stacks.

Supplemental References

Amir-Ahmady, B., Boutz, P.L., Markovtsov, V., Phillips, M.L., and Black, D.L. (2005). Exon repression by polypyrimidine tract binding protein. RNA *11*, 699-716.

Boutz, P.L., Stoilov, P., Li, Q., Lin, C.-H., Chawla, G., Ostrow, K., Shiue, L., Ares, M., and Black, D.L. (2007). A post-transcriptional regulatory switch in polypyrimidine tract-binding proteins reprograms alternative splicing in developing neurons. Genes & Development *21*, 1636-1652.

Buske, F.A., Boden, M., Bauer, D.C., and Bailey, T.L. (2010). Assigning roles to DNA regulatory motifs using comparative genomics. Bioinformatics *26*, 860-866.

Chen, W.V., Alvarez, F.J., Lefebvre, J.L., Friedman, B., Nwakeze, C., Geiman, E., Smith, C., Thu, C.A., Tapia, J.C., Tasic, B.*, et al.* (2012). Functional significance of isoform diversification in the protocadherin gamma gene cluster. Neuron *75*, 402-409.

Corbin, J.G., Gaiano, N., Juliano, S.L., Poluch, S., Stancik, E., and Haydar, T.F. (2008). Regulation of neural progenitor cell development in the nervous system. J Neurochem *106*, 2272-2287.

Friedman, B.A., and Maniatis, T. (2011). ExpressionPlot: a web-based framework for analysis of RNA-Seq and microarray gene expression data. Genome Biol *12*, R69.

Gogolla, N., Galimberti, I., DePaola, V., and Caroni, P. (2006). Preparation of organotypic hippocampal slice cultures for long-term live imaging. Nat Protoc *1*, 1165-1171.

Hill, M.O. (1973). Diversity and evenness: a unifying notation and its consequences. Ecology, 427-432.

Hollander, M., and Wolfe, D.A. (1973). Nonparametric statistical methods (New York,: Wiley).

Holzmann, H., and Vollmer, S. (2008). A likelihood ratio test for bimodality in two-component mixtures with application to regional income distribution in the EU. Asta-Adv Stat Anal *92*, 57-69.

Hoppe, A.D., and Swanson, J.A. (2004). Cdc42, Rac1, and Rac2 display distinct patterns of activation during phagocytosis. Mol Biol Cell *15*, 3509-3519.

Huang, D.W., Sherman, B.T., and Lempicki, R.A. (2009). Systematic and integrative analysis of large gene lists using DAVID bioinformatics resources. Nat Protoc *4*, 44-57.

Hubbard, K.S., Gut, I.M., Lyman, M.E., and McNutt, P.M. (2013). Longitudinal RNA sequencing of the deep transcriptome during neurogenesis of cortical glutamatergic neurons from murine ESCs. F1000Research *2*.

Jerabek, S., Merino, F., Scholer, H.R., and Cojocaru, V. (2014). OCT4: Dynamic DNA binding pioneers stem cell pluripotency. Bba-Gene Regul Mech *1839*, 138-154.

Kaech, S., and Banker, G. (2006). Culturing hippocampal neurons. Nat Protoc *1*, 2406-2415.

Khandelia, P., Yap, K., and Makeyev, E.V. (2011). Streamlined platform for short hairpin RNA interference and transgenesis in cultured mammalian cells. P Natl Acad Sci USA *108*, 12799-12804.

Liu, P., Jenkins, N.A., and Copeland, N.G. (2003). A highly efficient recombineering-based method for generating conditional knockout mutations. Genome Res *13*, 476-484.

Makeyev, E.V., Zhang, J., Carrasco, M.A., and Maniatis, T. (2007). The MicroRNA miR-124 promotes neuronal differentiation by triggering brain-specific alternative pre-mRNA splicing. Molecular cell *27*, 435-448.

Menezes, J.R., and Luskin, M.B. (1994). Expression of neuron-specific tubulin defines a novel population in the proliferative layers of the developing telencephalon. J Neurosci *14*, 5399-5416.

Miller, J.E., and Reese, J.C. (2012). Ccr4-Not complex: the control freak of eukaryotic cells. Crit Rev Biochem Mol Biol *47*, 315-333.

Mullen, R.J., Buck, C.R., and Smith, A.M. (1992). NeuN, a neuronal specific nuclear protein in vertebrates. Development *116*, 201-211.

Nagy, A. (2003). Manipulating the mouse embryo : a laboratory manual, 3rd ed edn (New York: Cold Spring Harbor Laboratory Press).

Orlandi, C., La Via, L., Bonini, D., Mora, C., Russo, I., Barbon, A., and Barlati, S. (2011). AMPA Receptor Regulation at the mRNA and Protein Level in Rat Primary Cortical Cultures. Plos One *6*.

Pevny, L.H., and Nicolis, S.K. (2010). Sox2 roles in neural stem cells. Int J Biochem Cell Biol *42*, 421-424.

Pollard, K.S., Hubisz, M.J., Rosenbloom, K.R., and Siepel, A. (2010). Detection of nonneutral substitution rates on mammalian phylogenies. Genome Res *20*, 110-121.

Qi, Y.P., Wang, J.K.T., McMillian, M., and Chikaraishi, D.M. (1997). Characterization of a CNS cell line, CAD, in which morphological differentiation is initiated by serum deprivation. J Neurosci *17*, 1217-1225.

Rasband, W. (1997). ImageJ. US National Institutes of Health, Bethesda, MD, USA.

Ray, D., Kazan, H., Cook, K.B., Weirauch, M.T., Najafabadi, H.S., Li, X., Gueroussov, S., Albu, M., Zheng, H., Yang, A.*, et al.* (2013). A compendium of RNA-binding motifs for decoding gene regulation. Nature *499*, 172-177.

Sambrook, J., Russell, D.W., Fritsch, E.F., and Maniatis, T. (2001). Molecular cloning : a laboratory manual, 3rd ed edn (Cold Spring Harbor, N.Y.: Cold Spring Harbor Laboratory Press).

Spellman, R., Llorian, M., and Smith, C.W.J. (2007). Crossregulation and functional redundancy between the splicing regulator PTB and its paralogs nPTB and ROD1. Molecular Cell *27*, 420-434.

Yap, K., Lim, Z.Q., Khandelia, P., Friedman, B., and Makeyev, E.V. (2012). Coordinated regulation of neuronal mRNA steady-state levels through developmentally controlled intron retention. Genes & Development *26*, 1209-1223.

Yuan, Q., Yang, F., Xiao, Y., Tan, S., Husain, N., Ren, M., Hu, Z., Martinowich, K., Ng, J.S., Kim, P.J.*, et al.* (2015). Regulation of Brain-Derived Neurotrophic Factor Exocytosis and Gamma-Aminobutyric Acidergic Interneuron Synapse by the Schizophrenia Susceptibility Gene Dysbindin-1. Biol Psychiatry.

Zheng, S., Gray, E.E., Chawla, G., Porse, B.T., O'Dell, T.J., and Black, D.L. (2012). PSD-95 is post-transcriptionally repressed during early neural development by PTBP1 and PTBP2. Nature neuroscience *15*, 381-388.
